# Qualitative study exploring the barriers to menstrual hygiene management faced by adolescents and young people with a disability, and their carers in the Kavrepalanchok district, Nepal

**DOI:** 10.1186/s12889-021-10439-y

**Published:** 2021-03-10

**Authors:** Jane Wilbur, Shubha Kayastha, Thérèse Mahon, Belen Torondel, Shaffa Hameed, Anita Sigdel, Amrita Gyawali, Hannah Kuper

**Affiliations:** 1 London School of Hygiene & Tropical Medicine, Keppel Street, London, WC1E 7HT UK; 2 Independent consultant, Kathmandu, Nepal; 3 WaterAid, 27-29 Durham Street, London, SE11 5JD UK

**Keywords:** Disability, Carers, Menstrual hygiene management, Water, Sanitation and hygiene, Rights

## Abstract

**Background:**

Menstrual hygiene management (MHM) is a recognised public health, social and educational issue, which must be achieved to allow the realisation of human rights. People with disabilities are likely to experience layers of discrimination when they are menstruating, but little evidence exists.

**Methods:**

The study aims to investigate barriers to MHM that people with disabilities and their carers face in the Kavrepalanchok, Nepal, using qualitative methods. Twenty people with disabilities, aged 15–24, who menstruate and experience ‘a lot of difficulty’ or more across one or more of the Washington Group functional domains were included, as well as 13 carers who provide menstrual support to these individuals. Purposeful sampling was applied to select participants. Different approaches were used to investigating barriers to MHM and triangulate data: in-depth interviews, observation, PhotoVoice and ranking. We analysed data thematically, using Nvivo 11.

**Results:**

Barriers to MHM experienced by people with disabilities differ according to the impairment. Inaccessible WASH facilities were a major challenge for people with mobility, self-care and visual impairments. People with intellectual impairments had difficulty accessing MHM information and their carers despaired when they showed their menstrual blood to others, which could result in abuse. No support mechanisms existed for carers for MHM, and they felt overwhelmed and isolated. Menstrual discomfort was a major challenge; these were managed with home remedies, or not at all. Most participants followed menstrual restrictions, which were widespread and expected; many feared they would be cursed if they did not. As disability is often viewed as a curse, this demonstrates the layers of discrimination faced.

**Conclusion:**

Issues related to MHM for people with disabilities is more complex than for others in the population due to the additional disability discrimination and impairment experienced. Research exploring these issues must be conducted in different settings, and MHM interventions, tailored for impairment type and carers requirements,should be developed. Attention to, and resourcing for disability inclusive MHM must be prioritised to ensure ‘no one is left behind’.

**Supplementary Information:**

The online version contains supplementary material available at 10.1186/s12889-021-10439-y.

## Background

Menstrual hygiene management (MHM) is a recognised public health, social and educational issue [1]. Research shows that the realisation of human rights is inhibited by lack of provision for MHM, including the right to education, health and work [2–7]. This can happen when there is: inadequate physical water, sanitation and hygiene (WASH) infrastructure to support menstruation at home and in public spaces, a lack of affordable, comfortable and appropriate menstrual products, a lack of accurate information on the menstrual cycle and how to manage it with dignity, as well as harmful social beliefs and taboos related to menstruation.

Underlying these issues is menstrual stigma, which is rooted in power and gender inequalities, and means that menstruation is not often openly discussed. This discourages sharing accurate information on the menstrual cycle, and how to manage it hygienically and with dignity [8]. It also leads people to be unsure how they can seek support at home, at school or through healthcare services [8]. This is the case in Nepal, where menstruation is not spoken about openly, and many pre-pubescent girls do not receive information about menstruation, so their first menstrual cycle can be a frightening experience [9, 10]. Accurate information on the menstrual cycle and how to manage it hygienically is likewise inadequate [11]. Management of menstrual discomfort is limited and menstrual hygiene information is predominantly shared between family members, and focuses on the use of menstrual products and maintaining the current social beliefs and taboos surrounding menstruation. In this context, unhygienic practices are common [9].

Cultural, religious and behavioural expectations related to menstruation varies globally and within countries, as does the extent to which these impact on people’s ability to fully participate in society when they are menstruating. A recent study in Nepal, found that 89% of women and girls experienced restrictions whilst menstruating [12], which involves the seclusion from the community and within the home [10, 13, 14]. Within Nepal, the extent to which menstrual restrictions are followed relates to caste and religion; 81.3% of the population are Hindu and adhere to the caste system, which is based on ritual impurity and purity [11, 15]. There are four broad castes: Brahmin, Kshatriya, Vaishya, Sudra, and these can determine individual’s behaviours, including how strictly menstrual restrictions are followed. For instance, Brahmins are the upper-caste, and tend to follow menstrual restrictions closely so that they are not contaminated by menstrual blood [12, 16, 17].

Evidence about how a lack of MHM negatively impacts gender equality is growing [1, 18, 19]. However, there is a consistent blind spot on disability, even though 15% of the world’s population have a disability, and 80% of people with disabilities live in low- and middle-income countries (LMICs) [20]. Where studies do exist, they focus on a specific impairment group such as intellectual, visual or mobility instead of gaining a holistic view of the barriers faced by people with different impairments [21–23]. Within South Asia, only one study explores the MHM experiences of people with intellectual impairments and their carers [22]. With a dearth of evidence on the MHM requirements of people with disabilities and their carers, very few interventions exist [5, 24].

This lack of attention is compounded by disability discrimination, demonstrated by misconceptions. These include that people with disabilities do not have the same reproductive systems as non-disabled people, so may not menstruate and cannot have children, or people with disabilities are considered contagious, dirty and impure [20, 25–27]. Without social assistance, carers struggle to support another person’s menstrual cycle [22, 28–30]. Management strategies applied include putting the person with a disability on long-term contraception, limiting their physical mobility during menstruation and sterilisation [22, 29–32]. Therefore, people with disabilities living in LMICs may face layers of discrimination when they are menstruating. These may negatively impact on the extent to which they can fulfil their human rights, including education, water and sanitation, and sexual and reproductive rights, but more evidence is required [5, 33, 34]. The study aims to begin filling this gap by investigating the barriers to MHM that adolescents and young people with a disability and their carers face in the Kavrepalanchok (Kavre) district, Nepal, using qualitative methods.

## Methods

### Research design

Phenomenological research methodology underpins this study, and influenced the data collection tools applied [35]. We recognise menstruation as a physiological and social phenomenon: a participant’s life experiences of menstruation are situated within socio-cultural factors, and menstrual related behaviours and opinions are shaped by individual and external influences. Our data collection methods guided participants to describe their lived experiences, beliefs and feelings about menstruation visually and verbally, and we observed the behaviour setting by carrying out accessibility and safety audits of the MHM facilities used. Interactions were reciprocal, and researchers answered participants’ questions about menstruation, providing accurate information on the menstrual cycle and how to manage it hygienically.

### Research team and training

Qualitative data was generated by the lead author (JW), a Nepali Research Coordinator (SK) and two Nepali Field Researchers (AS and AG). As we are committed to disability-inclusive research, we recruited AS (with a visual impairment) and AG (who has a mobility impairment) as field researchers and JW and SK mentored them throughout the data collection so that they could develop their research skills. All the research team members were women.

The research team participated in a week-long training, led by the lead author and a second experienced qualitative researcher on how to conduct research ethically with people who have a disability and their carers [36]. Participants included eight people with a range of disabilities, sign language interpreters, SK, and representatives from WaterAid, CBM and Plan Nepal. Attendees were involved in this study, and the ‘Strengthening voices of adolescents with disabilities in Nepal’ research, which aimed to understand what was important for the wellbeing of adolescents with disabilities [37]. The latter was conducted by the London School of Hygiene and Tropical Medicine (LSHTM), CBM and Plan International and started soon after the research training workshop. Participatory methods, including the Age Line [38], Feeling Dice [39], the Johari Window [40] and Collage [41], were applied to facilitate discussions on seeking informed consent, MHM, and confidently but sensitively discussing ‘private’ topics [42]. Guidance was given on how to generate data with people who have different impairments using qualitative methods, including ensuring breaks are regularly offered to participants throughout the process, always addressing the person with a disability directly and not an interpreter or carer, being encouraging, patient and respectful at all times. Specifically, when interviewing people with a hearing impairment, ensuring sign-language interpretation is available if required, speaking slowly, clearly and at a steady rhythm and never shouting. When interviewing people with visual impairments, identifying yourself clearly and introducing everyone in the room; not leaving when the person is talking, and asking the participant if they prefer to sit in bright light or shade as this may affect their vision. Researchers should sit at the same level as a person with a mobility impairment, and not push a wheelchair without asking the person first. For people with intellectual impairments, carers should be present; researchers should always acknowledge the participant’s contribution, speak in short, simple sentences and explain more than once if the person does not understand. Researchers should also be guided by the person’s body language and end the interview if the participant becomes disengaged. Data collection tools used in this study were tested and refined during this training.

### Study site

The Kavre district was selected as the study site as the research partner, WaterAid implements MHM programmes there with local NGOs, Karnali Integrated Rural Development and Research Centre (KIRDAC) and Centre for Centre for Integrated Urban Development (CIUD). The Kavre district is one of Nepal’s 77 districts, with a population of 381,937, it is classified as ‘mid-hilly’ [43]. The district’s basic water coverage is 89% and basic sanitation coverage is 98% (unpublished data). The Kavre district was the epicentre of the 2015 earthquakes and much of its infrastructure was destroyed, including household latrines. Efforts to rebuild infrastructure are ongoing.

### Study population and sample size

The study population and inclusion criteria comprised:

1. 20 individuals, aged between 15 and 24 years, who menstruate and experience‘a lot of difficulty’, or more across one or more of the visual, hearing, mobility, cognition, self-care and communicationfunctional domains [44].
2. 13 carers, who were selected if they provided menstrual care for a participant with a disability. Carers were interviewed about their experiences of supporting another person’s menstrual cycle, as well as their interpretation of the person with disability’s MHM experiences and related feelings.

Table 1. details the study population characteristics, including ages, locations and functional domains.

**Table 1:** **Study population characteristics**

Study population	Variables	n=
Person with a disability	*Age group*	
	15–19	9
	20–24	11
	*Location*	
	Urban	16
	Rural	4
	*Functional domain*	
	Visual	3
	Hearing	2
	Mobility	8
	Cognition	1
	Self-care	1
	Multiple*	5
Carer	*Location*	
	Urban	6
	Rural	7
	*Functional domain of the person with a disability*	
	Visual	1
	Mobility	3
	Cognition	7
	Multiple*	2
	*Person providing care*	
	Relative	11
	Professional	2

*Multiple included: mobility, cognition, self-care and communication

We applied purposeful sampling to select participants who experience the phenomenon researched [45, 46]. Firstly, the lead author explained the Washington Group short set of questions [25] to WaterAid’s partner organisations: KIRDAC, CIUD and government social mobilisers, who had knowledge of people with disabilities living in the study area. The Washington Group Short Set, is a tool recommended for data disaggregation, and includes questions about a person’s abilities across six functional domains: visual, hearing, mobility, cognition, self-care, and communication [44]. Disability is identified as anyone having at least ‘a lot of difficulty’ in one or more of these domains. Functional domains are referred to as ‘impairments’ in this article, and cognition as an ‘intellectual impairment’.

These representatives identified 20 females with a disability, aged between 15 and 24. Secondly, the research team visited the potential participants and asked them the Washington Group short set of questions [25], their age and if they menstruate to confirm that they met the inclusion criteria. Participants that did not qualify were excluded. We intentionally selected people with a range of impairment types, so we applied snowball sampling to ensure representation across the six functional domains. Snowball sampling is a method of increasing the sample by asking participants to identify other people to interview [47, 48]. Carers were selected if they provided menstrual care for a participant with a disability.

### Data collection methods and activities

Data collection was carried out in September 2017. The study applied four different qualitative methods to allow for methods triangulation: In-depth interviews, observation and PhotoVoiceand ranking (described in Table 2) [50]. Additional Files 1-3 contain the in-depth interview guide for carers and people with disabilities, and PhotoVoice guidance, which were developed for, and used in this study. PhotoVoice participants were selected after their in-depth interview; they represented different impairments (mobility, self-care) and roles (person who menstruates, or supports another’s menstrual cycle), settings (household and residential institution) and spoke about their menstrual experiences openly. PhotoVoice can be a very effective method for a person to represent their experiences visually, but it can be time intensive and takes approximately one day to complete. We aimed to complete PhotoVoice with four participants with a disability and two carers, but two individuals declined.

**Table 2:** Summary of methods

Method	Purpose	Description	Sample characteristics	Sample size
In-depth interview	To understand barriers to MHM, and how these effect participants’ lives.	Undertaken at the participant’s home, school, care home or hospital; interviews lasted between 1 and 1.5 h. With consent, interviews were conducted in Nepali, recorded on a voice recorder, and translated into English if JW (who does not speak Nepali) was present. Field notes were made after the interviews.	Individuals with disabilities, aged 15–24 and menstruates, and carers who support them	20 individuals with disabilities 13 carers
	To understand the menstrual products available, user preference and rationale.	Market survey, product attribute assessment and user preference with ranking: a range of menstrual products available on the local market were displayed to participants during interviews. Researchers asked participants if they had used any, their preference with reasons for why, and to rank the products according to preference. A photo was taken of the products in ranked order.	Individuals with disabilities	16 individuals with disabilities
Observation	To observe if any participants face accessibility or safety barriers when using water, sanitation and menstrual hygiene management facilities (revised version of *WEDC, WaterAid (2013) Accessibility and safety audit* [49]).	Observed participants demonstrating where they stored their menstrual product, how they changed, washed and/or disposed of it; where they collected water from, what soap was used, and where they washed their bodies. Issues explored included accessibility, privacy and safety of facilities, such as distance to water source, ability to use facilities independently, if the participant can be seen in the facility; if it is well lit, if it has a door with a lock that can be used independently. Photographs of facilities were taken and field notes recorded. Observation took place after in-depth interviews.	Individuals with disabilitiesand carers	20 individuals with disabilities 13 carers
PhotoVoice	To allow participants to express themselves visually; allow participants time to reflect on the issues, and rank their experiences against perceived levels of importance.	Cameras were lent to participants, who were asked to take five photos of their own menstrual experience or of caring for another person’s menstrual cycle. Photos were printed and discussed with the participants, who provided captions and ranked the photos according to which was the greatest to least important issue. The whole process took 0.5 to 1 day per participant. All participants requested that their real names are credited whenever their photos and captions are used. Participant’s names are used in this article.	Individuals with disabilities, able to understand the task, use a camera or direct a third party to take photos. Carer who provides care throughout the menstrual cycle.	3 individuals with disabilities 1 carer

At the end of each day, the research team met to discuss findings and themes; when all topics were explored and no new data emerged, we concluded that data saturation was met and stopped data collection.

### Data analyses

Data analyses was iterative: the research team met at the end of each day to review field notes, discuss their influence within the data collection process, and emerging themes (such as similarities of barriers to accessing MHM facilities for people with mobility impairments, or a lack of support for carers of persons with disabilities), as well as to review and revise the interview technique and topic guides. When data collection was complete, voice recordings of interviews were translated and transcribed verbatim into English. Transcriptions were checked for accuracy by Nepali researchers in the team and WaterAid Nepal staff. Transcripts were not returned to participants for comment or correction, but overall research findings were shared with participants at a later date (see Wilbur and Bright, 2019 [24]).

The lead author then applied a thematic analytical approach to analyse transcriptions, which involved: 1) familiarisation with the data, 2) generation of initial codes that were structured according to outcomes in the adapted socio-ecological framework for menstrual hygiene management [5]. Outcomes included: access to water and sanitation facilities, including menstrual materials disposal mechanisms, appropriate menstrual materials, relationship with carers / the person with a disability, ability to manage menstruation independently, ability to manage another person’s menstrual cycle and tasks carried out. 3) Searching for, and identifying sub-themes within the outcomes included in the adapted socio-ecological framework for MHM, 4) reviewing sub-themes, 5) defining and naming sub-themes and 6) producing the report [51]. Codes were compared and relationships between codes were identified and analysed using analytic memos in NVivo 11.

## Results

In summary, results showed that:the barriers to MHM differ depending on the person’s impairment, disposable menstrual pads were preferable, but disposal practices and services were inadequate, pre and menstrual symptoms were not well understood or managed; menstrual restrictions added additional layers of challenges for people with disabilities and carers, and that there was inadequate menstrual hygiene information, training and support.

### The barriers to MHM differ depending on the person’s impairment

People’s experience of managing menstruation was influenced by their impairment. For instance, people with mobility impairments identified challenges related to their use of the menstrual product. Some reported that the type and positioning of the product made it uncomfortable to sit in a wheelchair all day. Many other participants with mobility impairments were concerned that the product was not absorbent enough and worried about leakages.

*"When I sit in the wheelchair the pads may fold or something like that might happen which makes me feel uneasy […]. It becomes very uncomfortable to sit. Unlike my sisters who keep moving around, I have to sit in a place continuously. I get angry then and it gets difficult”*(person with multipleimpairments).

Inaccessible WASH facilities affected people with mobility impairments most severely. They were unable to easily or safely reach the place they change their menstrual product, comfortably change, wash or dry their menstrual product, or wash their bodies in private.

*“[I] need to wash the menstrual cloth in the toilet. There is no water in the toilet…. I have to carry water in a bucket while also managing the crutches […] I can’t wash [the menstrual] cloths either. [……] I keep it under my bed when I can’t wash it, and wash it when I get water. I have a problem during menstruation when there is no water”* (person with a mobility impairment)*.*

*“For those with spinal cord injury, it is easier and necessary for them to use this kind of [raised, Western] toilet. During their period they can’t stand to change their pads so these kinds of toilet become more essential”* (person with a mobility impairment)*.*

**Fig. 1:**
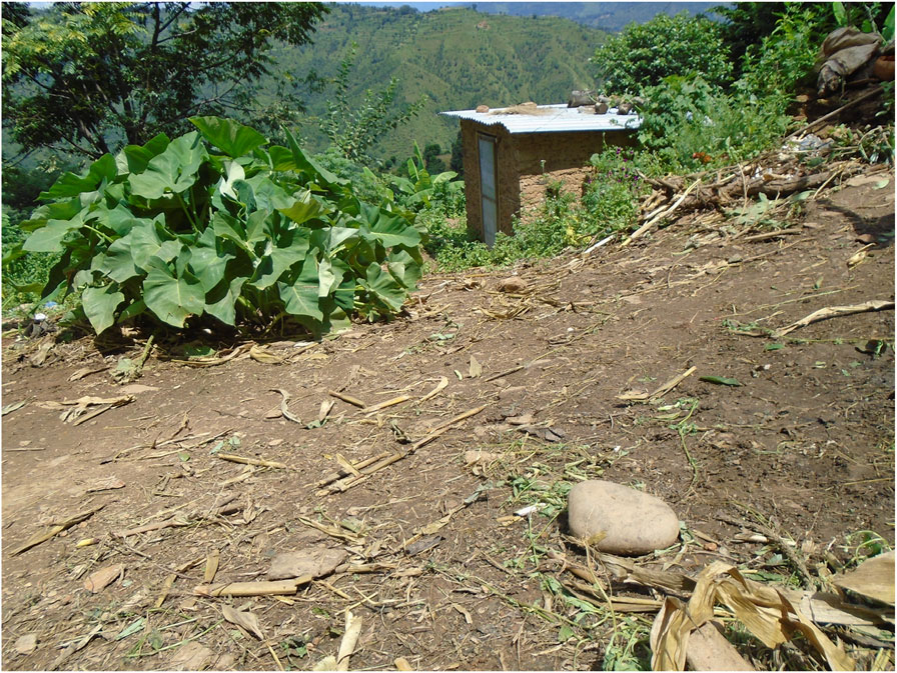
“It is difficult to go to the toilet.” PhotoVoice image taken by Sharmila Tamang. Ranked 1 out of 4

**Fig. 2:**
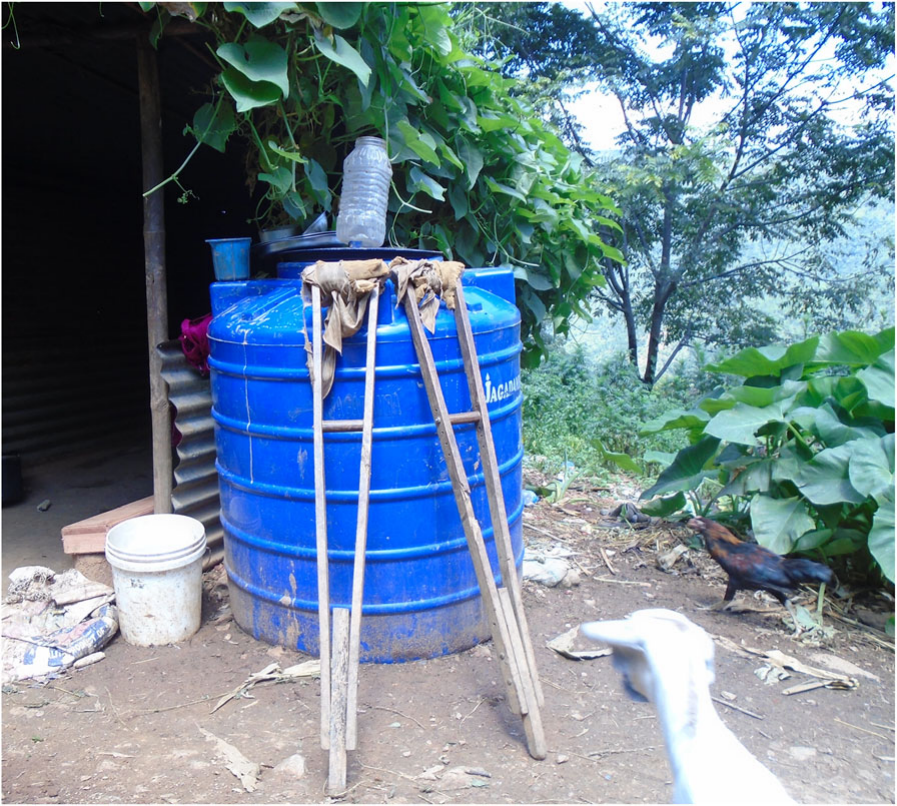
“Water issue is also there, I would have to carry water which is difficult.” PhotoVoice image taken by SharmilaTamang. Ranked 2 out of 4.

**Fig. 3:**
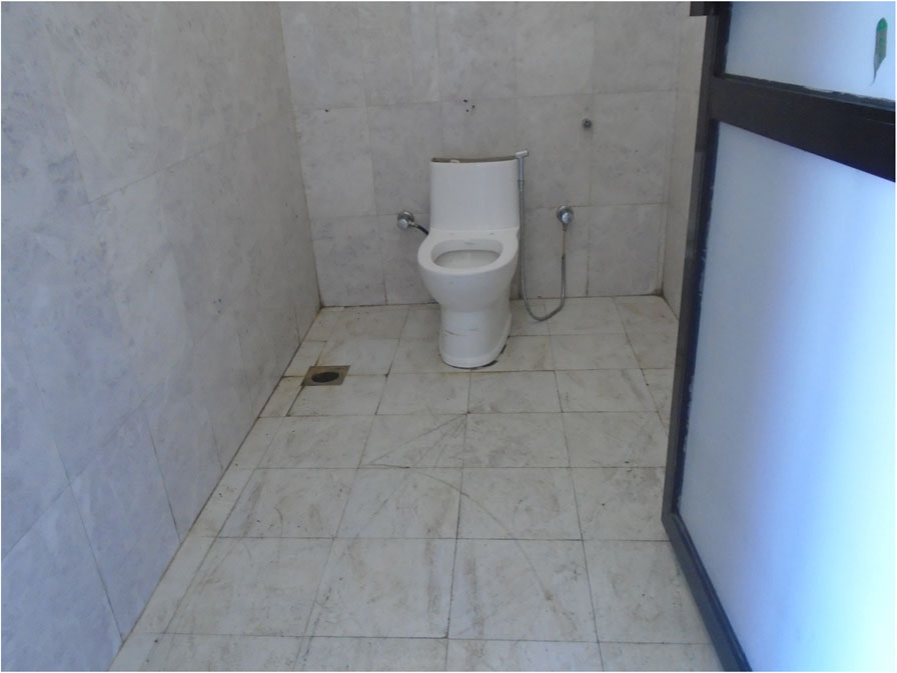
“It is not only easier to use this type of toilet for those with spinal cord injury but it is a necessity. So even in villages, these types of toilets should be built for people as not everyone can live in the cities. During period also it is difficult to stand to change pads so these kind is easier to use.” PhotoVoice image taken by Babita Thapa. Ranked 1 out of 5

**Fig. 4:**
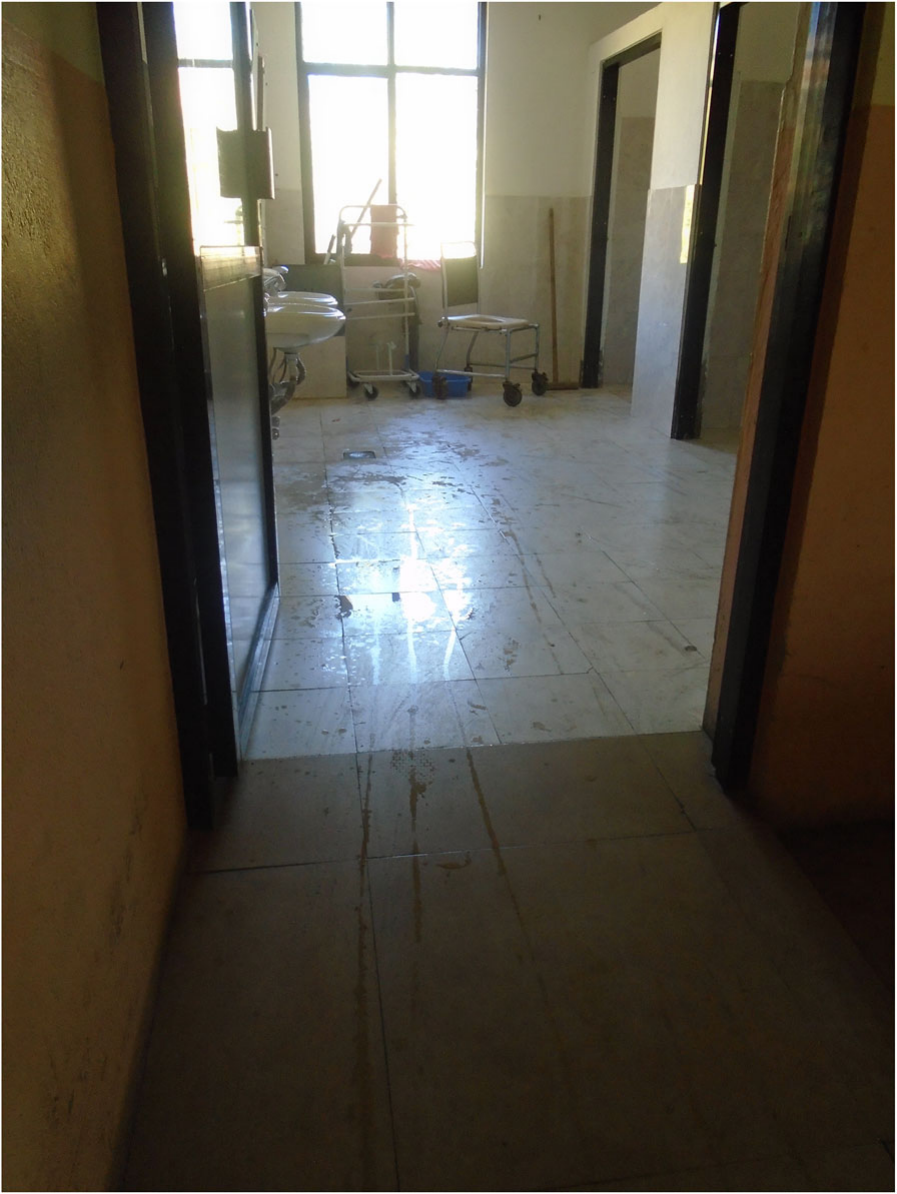
“During period one should be careful using bathroom. Our legs might already feel weak so we might fall down and meet an accident. Here the toilets are made for everyone to use but if it was to built at home for crutches user, it is to be made in a way that it is not slippery. Marble should not be used as it is slippery.” PhotoVoice image taken by BabitaThapa. Ranked 3 out of 5

**Fig. 5:**
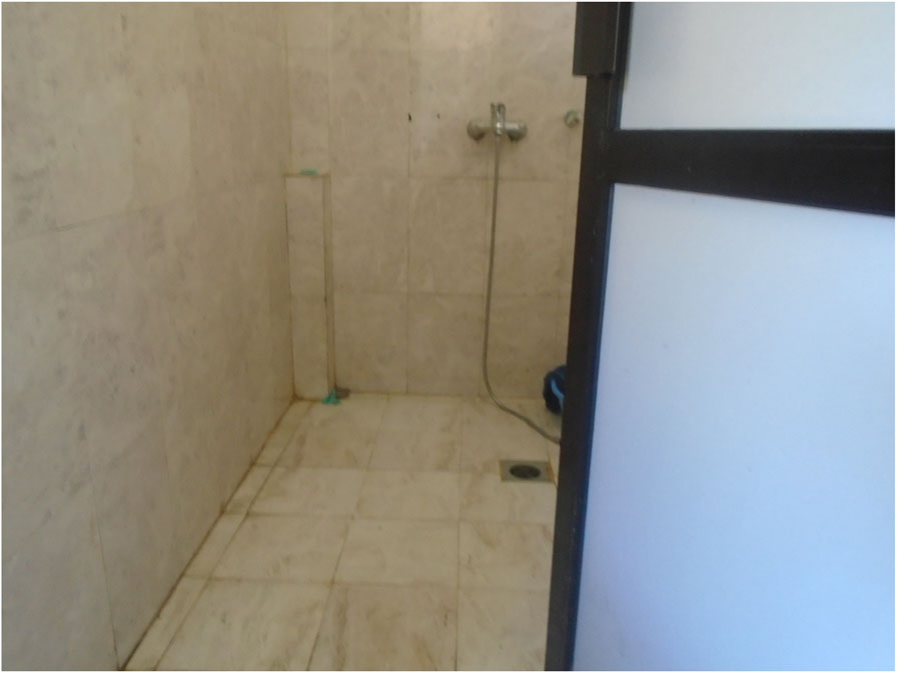
“For us to be able to wash our own clothes, bathroom should be made in such a way that we can wash our clothes ourselves while sitting on wheelchair. It will be much easier. I can’t wash clothes while standing or sitting. At home, I sit in a small stool but there is none here. If the washing space could be reached while sitting on a wheelchair, it would be good.” PhotoVoice image taken by Babita Thapa. Ranked 5 out of 5

**Fig. 6:**
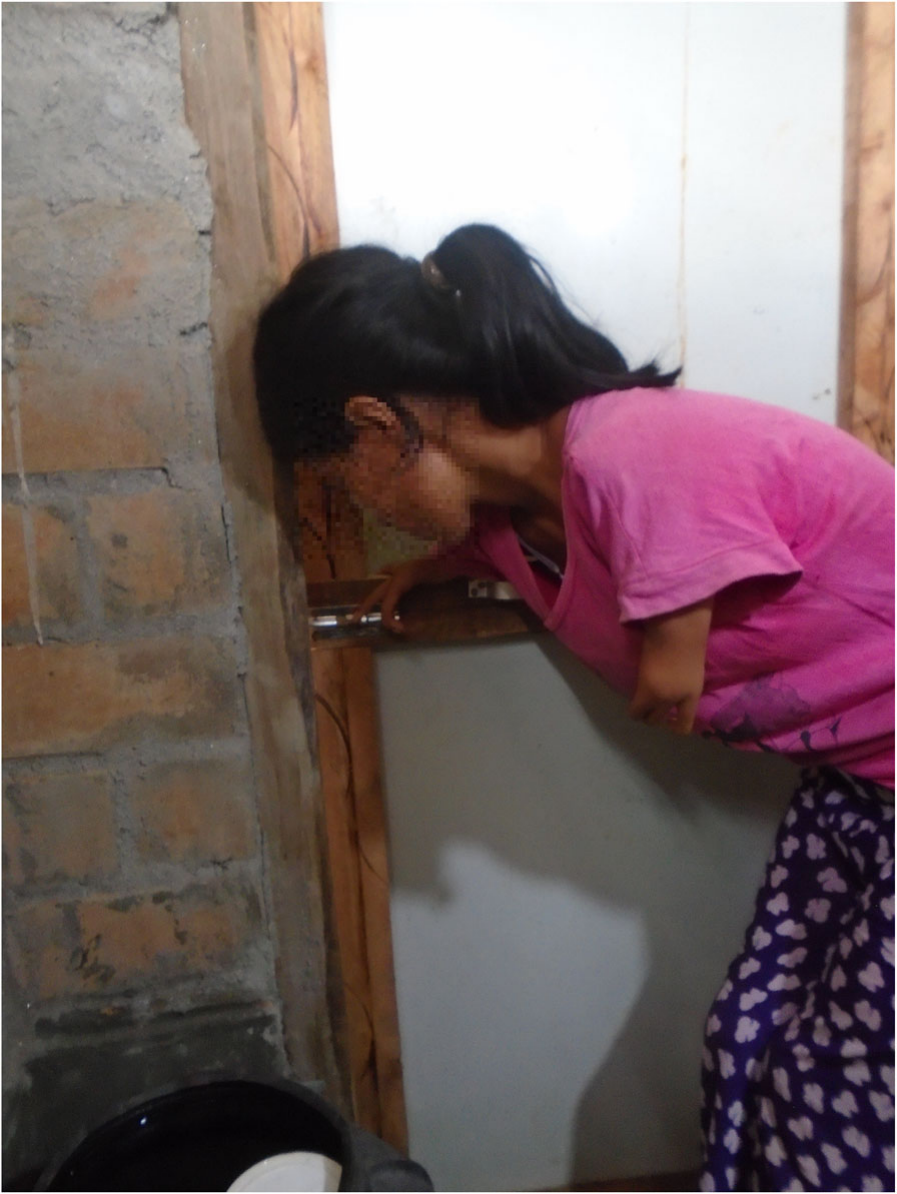
“When I have to use the toilet, I need someone else to help with the latch otherwise I can’t do it myself.” PhotoVoice image taken by Tulasa Karki. Ranked 3 out of 4

All of the people with disabilities who participated in PhotoVoice took images to show how inaccessible WASH facilities presented a major challenge. Of the nine photos taken by Sharmila and Babita (who have mobility and/or self-care impairments), five images related to inaccessible WASH facilities (Figs. 1, 2, 3, 4 and 5, Additional file 4: PhotoVoice images). Tulasa, who has a self-care impairment, took four photos and one related to a lack of safe and private WASH facilities (Fig. 6, Additional file 4: PhotoVoice images).

Carers reported that some participants with intellectual impairments, had difficulties retaining MHM information. Carers repeatedly told them how to change and wash the menstrual cloth every month and every time it needed changing, but found this frustrating.

People with visual impairments highlighted difficulties seeing blood on clothes and bed sheets, and disposing of the product discretely. This was stressful and worrying because of the prevailing menstrual taboos:

*“While washing the pants we know which parts to wash properly, but with the bed sheets, I cannot see the stains so it is difficult for me to clean the stains properly[….]. For throwing the pads in the dustbin, sometimes the dustbin may be outside the toilet, so I might have to throw it outside of the toilet. At those times I feel worried that a male person from my family would see them* (person with a visual impairment).

Participants with self-care impairments, who relied on carers, felt humiliated when asking another person to change their menstrual product, and guilt seeing their carer handle their menstrual blood. Consequently, they changed their menstrual product less frequently than they wanted to.

*“She says that the blood smells during my periods. [….]. She finds it disgusting. […]. I feel bad. If I had my own hands, I wouldn’t suffer so much. I wouldn’t have to depend on someone else. I could do it on my own; it’s not something you show it to others. I feel like crying. I feel bad”* (person with self-care impairments).

*“Even to change a pad I have to wait until my sister comes in the evening and helps me change, if not, I will have to wear the same pad till tomorrow”* (person with self-care impairments).

In contrast, people with hearing impairments interviewed said they did not face any specific challenges explicitly related to their disability.

### Disposable menstrual pads are preferable, but disposal practices and services are inadequate

All participants had access to menstrual materials, including commercial pads, menstrual cloth or tailor made pads, and results from the menstrual product market survey show that preference is highly individualised. Results of the market survey, product attribute assessment and user preference, show that disposable commercial pads with wings were preferred, and cloth was the least preferred. Table 3 captures the results across 16 people with a disability.

**Table 3:** Markey survey: most and least preferred menstrual product

Menstrual product	Preference	
	Most preferred	Least preferred
Disposable commercial pad with wings	8	0
Disposable commercial pad without wings	2	3
Cloth	3	5
Reusable tailor-made pad with wings	3	4
Reusable tailor-made pad without wings	0	0
Diaper	0	1
**Total**	16	13

Though the disposable commercial pads with wings were preferred by participants, hygienic and environmentally friendly disposal behaviours were often inadequate. Many participants threw used disposable commercial pads in rivers or down hillsides, so other people were less like to see the used product. Some wrapped the pads in plastic, so they were less visible. These behaviours did not correlate to impairment type; instead, reasons include a lack of waste disposal options and little knowledge about the environmental consequences.

**Fig. 7:**
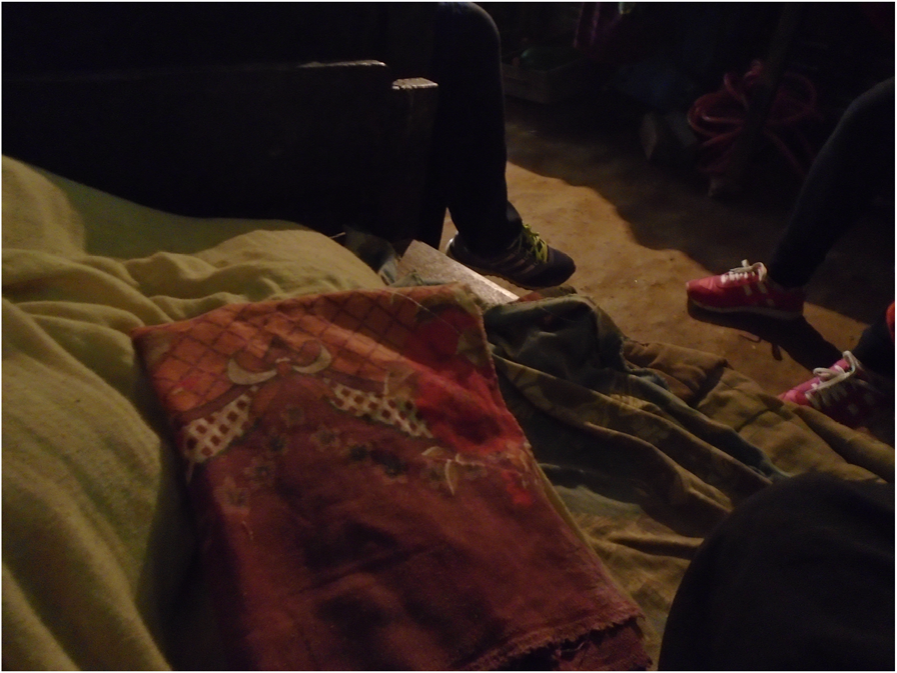
“Pad is easy to use compared to cloth.” PhotoVoice image taken by Sharmila Tamang. Ranked 3 out of 4

There were no clear preferences for product type by impairment due to the small number of participants in each category (see Tables 1 and 2 in Additional file 5: Menstrual product preference across impairment type). Through PhotoVoice, Sharmila (who has a mobility impairment) explained that she prefers using disposable commercial pads as they do not require washing, which she finds particularly challenging as she is unable to carry water and use her crutches (Fig. 2 and Fig. 7, Additional file 4: PhotoVoice images).

### Pre and menstrual symptoms are not well understood or managed

Many participants said that menstrual cramps were one of the biggest challenges they face when menstruating. Pain management strategies included home remedies, such as drinking warm water, sleeping and tying a cloth tightly around the abdomen. There is a belief that commercial pain relief tablets can damage your health, so few people took them and few carers provided them.

*“If I take medicine I will have more pain during the next period. That’s why I don’t take any medicine”* (person with a mobility impairment).

Carers reported that changes in behaviour before and during menstruation for participants with intellectual impairments included withdrawal, increased hyperactivity, self-injury, showing their used menstrual product to others, excessive sleeping, being frightened, withdrawn and refusing to eat. Without social support mechanisms to help people understand and respond to the changes in behaviour, carers of people with intellectual impairments felt frustrated and overwhelmed, as demonstrated by these quotes.

*“When she gets her period, if I ask her if she wants to put [a menstrual] cloth [on], she would just go to her room” *(carer of a person with an intellectual impairment)*.*

*“She doesn’t understand, she won’t listen. [….]  For someone like my daughter who does understand but wouldn’t remember, we can’t do much”* (carer of a person with an intellectual impairment)*.*

*“I feel annoyed. She doesn’t listen to me” *(carer of a person with an intellectual impairment)*.*

R: “Have you ever spoken to any medical people about her menstruation?”

C: “What could we do! We can’t stop it.”

### Menstrual restrictions add additional layers of challenges for people with disabilities and carers

Most participants followed menstrual restrictions, which dictate that menstruating people must sleep separately, are not allowed to worship, enter the kitchen, cook or touch plants, because it is believed that menstrual blood is dirty and contaminating.

*“Dirty blood leaves the body during period, so we should not worship during that time”* (person with a hearing impairment).

**Fig. 8:**
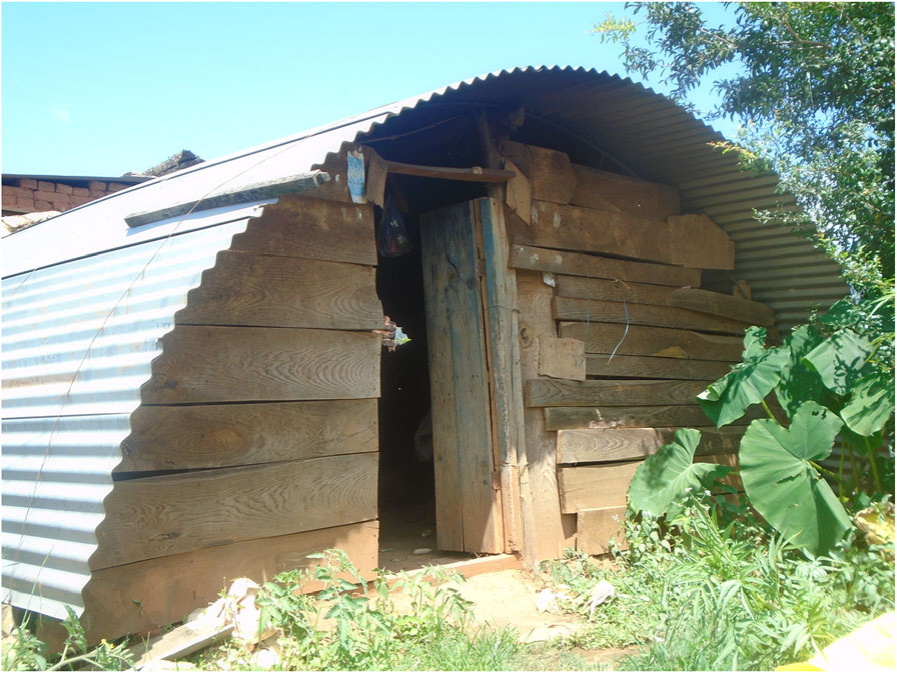
“During menstruation we are not allowed to enter the house.” The image is of the hut Tulasa sleeps in when menstruating. PhotoVoice image taken by Tulasa Karki. Ranked 2 out of 4

**Fig. 9:**
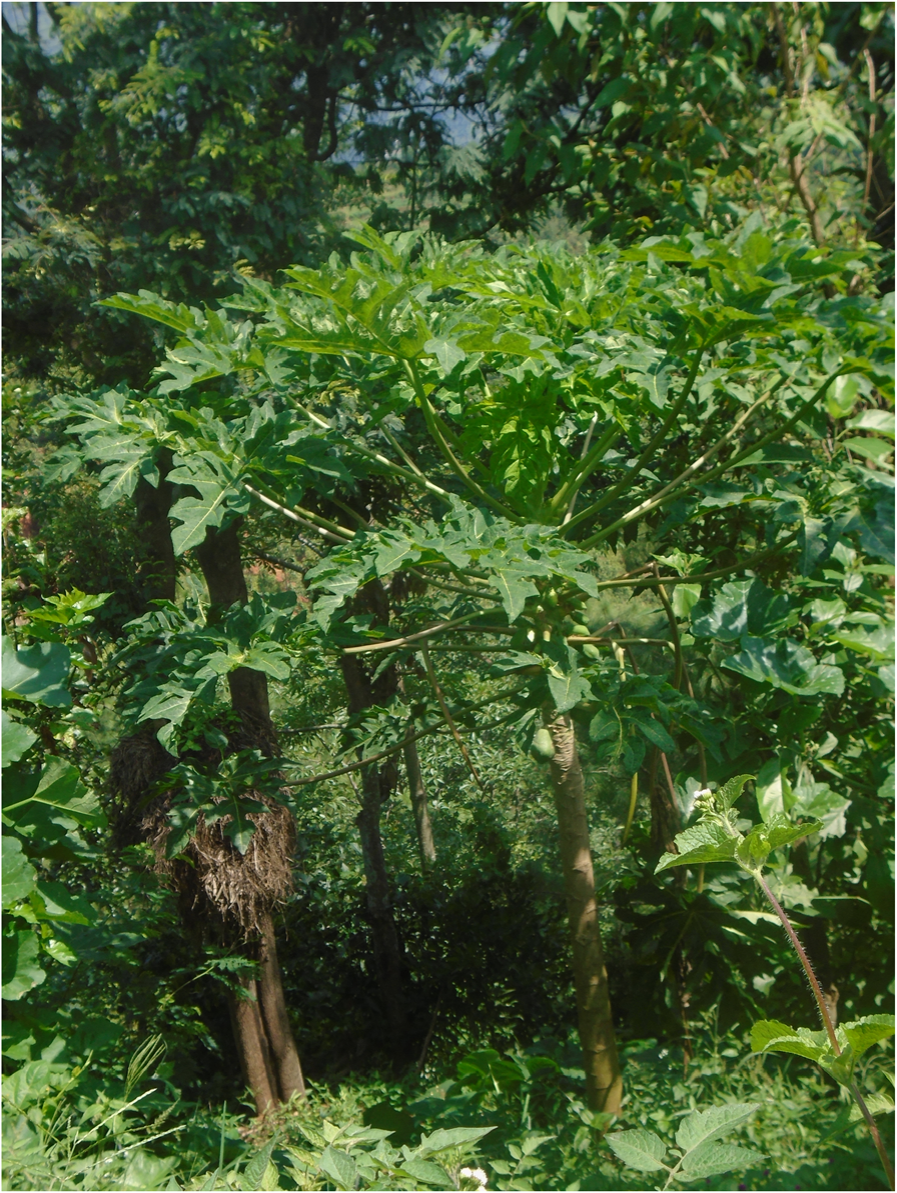
“There is a belief that if you touch plants during period, it will rot.” PhotoVoice image taken by Tulasa Karki. Ranked 4 out of 4

Tulasa took two photos representing the menstrual restrictions: the hut, outside her home where she sleeps when menstruating, and plants that must not be touched during menstruation (Figs. 8 and 9, Additional file 4: PhotoVoice images). During an in-depth interview, one participant also explained she lived in a cow shed during menstruation when at home.

*“I had to be banished in the cowshed for seven to 12 days”* (person with ahearing impairment).

If a person does not adhere to the restrictions, it is believed that the gods will curse the family. Disability was also viewed as a curse. Therefore, people feared that they will be doubly cursed if they did not follow restrictions.

*“….I am already suffering like this and people say that my disability is a curse, so if I don’t obey I will be further cursed”* (person with a mobility impairment).

People with visual impairments reported that menstrual restrictions were a major source of concern, fearing that they might inadvertently touch a ‘restricted object’, and thus lead the gods to curse the family.

*“I also cannot go against my family. It has been followed by our family, it is a tradition. […] I feel odd to move around because I am worried that I might touch [things that I should not]”* (person with a visual impairment)*.*

**Fig. 10:**
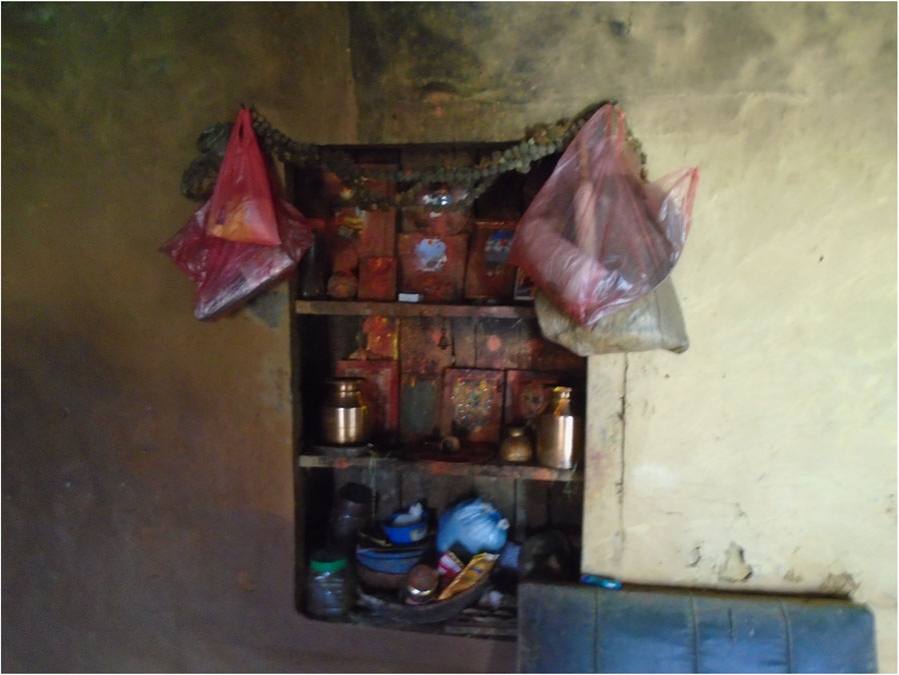
“Cannot touch during period as per our tradition.” The image is of worshiping area inside Bishnu’s home. PhotoVoice image taken by Bishnu Maya Sapkota. Ranked 1 out of 4

**Fig. 11:**
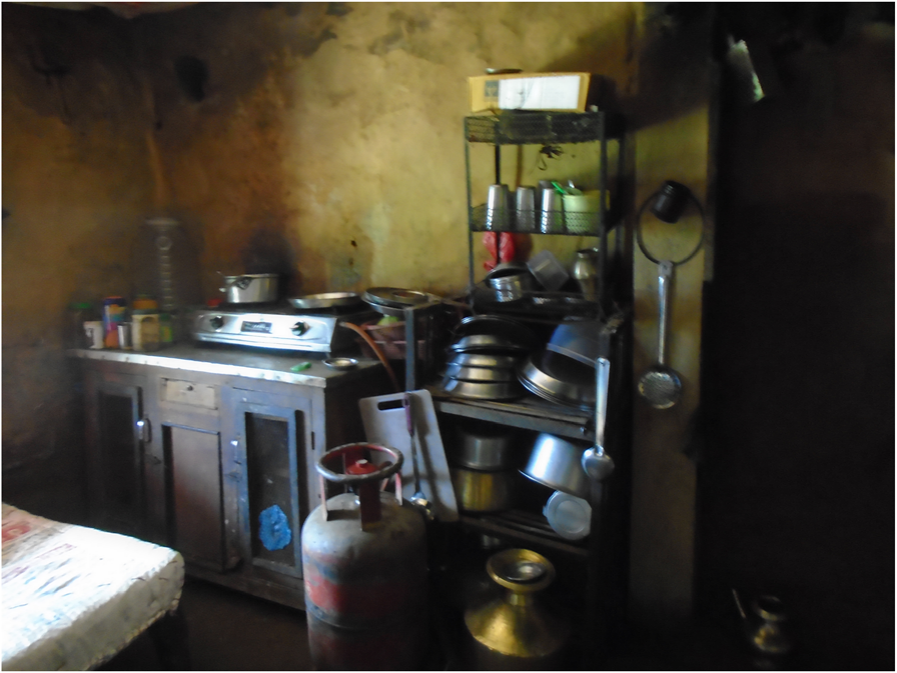
“We have to follow our tradition, so should not touch kitchen during period. If one touches kitchen, worshipping area is also touched. If touched, I feel discomfort and fear that something might happen. However, in case of my daughter if she touches, god will forgive her.” PhotoVoice image taken by Bishnu Maya Sapkota. Ranked 2 of 4

Two of four PhotoVoice images taken by Bishnu (a carer of a person with an intellectualimpairment), focused on menstrual restrictions (Figs. 10 and 11, Additional file 4: PhotoVoice images). She ranked these as the biggest challenges she faces when her daughter menstruates. Similarly, during in-depth interviews, carers of people with an intellectual impairment reported being worried that their family would be cursed if restrictions were not followed. Additionally, carers of people with an intellectual impairment explained some people did not wear a menstrual cloth, preferring to soak up the menstrual blood with underwear or trousers. Some participants isolated themselves when they are menstruating and otherswent out with blood stained clothes. One carer explained*:*

*“She just walks like that with blood on her clothes”* (Carer of a person with multiple impairments).

*“She would take it out and show it to others and would tell them to look at it. It was embarrassing”* (Carer of a person withan intellectualimpairment).

### Inadequate menstrual hygiene information, training and support

Some carers were surprised when their charges reached menarche, and one carer did not believe it when the person with a disability said they were bleeding until they saw the menstrual blood. Furthermore, information on menstrual hygiene was commonly withheld from people with intellectual impairments, though one carer did persist and explained that her charge *“took one year to understand the process and experience”* (carer of a person with multiple impairments). One carer reported that a teacher sent her daughter home from school at the onset of menarche, and the carer never sent her back.

*“That day, [her] teacher showed up at the house and suggested not to send [her] to the school because [she] had her mensuration in the classroom and the blood leaked on the bench she was sitting on. They said that it is difficult for [her] to take care of herself during the menstruation so it would be better that she stays at home and we take care of her. Then I stopped sending her to the school”* (carer of a person with multiple impairments).

Providing menstrual care was viewed as a very private issue by carers: very few discussed this subject with other people, including medical professionals. No support or support networks existed for carers, and many carers felt isolated and overwhelmed.

*“We don’t know anything else. I don’t go anywhere. I hear that people come to our village to teach about those things, but I haven’t been taught about menstruation management” *(carer of a person with multiple impairments)*.*

Two carers of people with a self-care impairment requested MHM training for the young person in order to increase the young person’s independence. A motivation for carers was fear for the future, as they worried about who would look after their daughter when they are no longer able to.

*“For now, I am here, but in future we don’t know what will be the situation. […]. I won’t live long but she has lots of time, I am very worried" *(carer of a person with multiple impairments).

## Discussion

This qualitative study among people with disabilities and their carers living in Nepal responds to the calls for information on the MHM barriers faced by people with disabilities, and contributes new evidence to the global discourse on MHM for the largest minority group [5, 52–55]. To the authors’ knowledge, this is the only study to date which investigates the barriers experienced across all impairments. We found that the barriers to MHM are complex and differ according to the person’s impairment. Furthermore, these barriers inhibit the person with a disability’s ability to live a dignified life and fulfil their human rights, including going to school.

### The barriers to MHM differ depending on the person’s impairment

To meet the MHM requirements of people living with a disability, water, sanitation and hygiene facilities must be located close to where the person lives. For instance, people with mobility, self-care and visual impairments may require an accessible water point inside latrines, accessible locks on toilet doors, raised toilet seats, non-slippery toilet floor, accessible washing and drying area for the body, clothes and menstrual products, as well disposal mechanisms that can be easily used by everyone.

Research exploring the barriers to MHM experienced by people with different impairments and their carers in different LMIC settings must be conducted to allow for comparison. Findings should be incorporated into the global MHM agenda alongside researching MHM for school going girls and non-disabled women [56–58]. Furthermore, MHM interventions appropriate for impairment type, and carers must be developed to overcome barriers.

Participants with hearing impairments in our study explained that they did not face challenges related to their disability. However, these participants all attended a school for children with hearing impairments and said they read about menstruation in books, and were supported by friends and teachers to practically manage menstruation. This finding may not reflect the experiences of people with hearing limitations who do not attend this type of school.

The study demonstrates that people with disabilities can be separated into those who manage their menstrual cycle independently, and possibly with great difficulty, and those who are reliant on carers for MHM. MHM interventions for people without disabilities are delivered directly to the person who menstruates. Yet, for people with disabilities, there may be a third party (carer) involved, who also requires MHM information and support so that they can help another person manage their menstruation comfortably, hygienically and with dignity. Such interventions must cover all aspects of MHM, including addressing harmful social beliefs, so that people who are reliant on carers to manage their menstrual cycle do not feel humiliated when asking for support, or guilty when carers change their menstrual material. These emotions are driven by shame and disgust caused by menstrual taboos. Carers must be supported to understand that supporting another person to manage their menstrual cycle as independently as possible would benefit both parties. Additionally, low cost lifting devices to support carers bathe and change a menstrual product should also be widely promoted to support carers and protect the person with disability’s dignity (see section 4.4 in Rosato-Scott et al. (2019) [59]. If carers move the person without such devices, they may experience back pain and associated issues [60].

### Disposable menstrual pads are preferable, but disposal practices and services are inadequate

More research is needed to identify comfortable, appropriate and affordable menstrual products for all people with disabilities, including people who are unable to sit out of bed unaided and / or who experience incontinence. Clear information on each product option, their implications for use and disposal need to be disseminated so that people can make informed choices. Policy makers and implementers should be encouraged to strengthen waste management service chains and incorporate menstrual waste management within it.

### Pre and menstrual symptoms are not well understood or managed

Menstrual discomfort was a key challenge expressed by participants and carers did not always manage this. A lack of pain management may have more negatively impacted people who have intellectual and / or communication impairments as they may not have understood the cause of the discomfort, or been able to communicate if they were in pain. This raises concerns that unmanaged pain may negatively impact on behaviours, which in turn can make carers feel overwhelmed, and a negative cycle forms. This is documented in three studies, which linked an inability of people on the autistic spectrum to understand the reason for menstrual discomfort or communicate when in pain. Menstrual related behaviours documented across this population in these studies included increased hyperactivity, self-injury, fatigue and anger [22, 28, 61].

### Menstrual restrictions add additional layers of challenges for people with disabilities and carers

Menstrual taboos, including restrictions to movements, are widely followed by people with and without disabilities in Nepal [12–14, 62]. However, due to vulnerability to violence, people with disabilities may be more susceptible to abuse when sleeping in menstrual huts [63–65]. For instance, a person with a hearing impairment may not hear an intruder approaching; people with a mobility impairment may be less able to escape, and people with communication and/or intellectual impairments may be less able to disclose experiences of abuse. Our study also highlights the fear expressed by people with disabilities that they would be ‘doubly cursed’ if they did not adhere to the menstrual restrictions. These findings reveal layers of stigma and discrimination faced by people with disabilities in Nepal when they menstruate.

Our findings show that carers were particularly concerned that the young person with an intellectual impairment would not follow cultural and social norms (including menstrual restrictions); that they would refuse to wear a menstrual product and would go out with menstrual blood on their clothes. In a study conducted in India, where the socio-cultural norms are similar to those of Nepal, carers reported the biggest challenge faced was that their daughters with an intellectual impairment would not wear a menstrual pad, and leave home with blood stained clothes. As a result, the carers would keep their daughters at home whilst they were menstruating, put them on long-term contraception or sterilise them [22]. When people with a disability in our study went out in public with blood stained clothes, community and family members verbally and physically abused them. Therefore, ensuring MHM for this population is also a safeguarding issue.

### Inadequate menstrual hygiene information, training and support

The misconception that people with disabilities do not have the same reproductive systems as non-disabled people [20, 26], means they are even less likely to receive MHM information than non-disabled people. Additionally, such information was commonly withheld from people with intellectual impairments, because of the perception that they would not understand it. However, consequences of providing repetitive and simple MHM information for one participant was a greater ability to manage her own menstruation. Thus, people with intellectual impairments may be able to understand information about the menstrual cycle, if it is tailored to their level of understanding and repeated regularly. Ability to recall information would be dependent on the extent of the intellectual impairment.

MHM information is mainly delivered at schools, but many participants with intellectual impairments did not attend school so were excluded from receiving this information. One participant was sent home from school at the onset of menarche, and this marked the end of her formal education, which could negatively impact on her life chances [66]. Carers who were unable to leave the home because of caring duties were also at a disadvantage as they were unable to access MHM information shared in the community.

Our study showed that there is a lack of social support and information about how to care for another person’s menstrual cycle, that menstrual care is viewed as a private issue, and that this results in carers feeling overwhelmed and isolated. These findings are reflected in studies from Taiwan, India, and the UK [22, 28, 29]. They showed that menstruation is viewed by carers (family members) as a confidential topic, so people did not speak to others or seek support from anyone, including medical professionals. This lack of support can negatively affect carer’s wellbeing [5, 22, 28, 67–69].

### Strengths and limitations

The strengths of this study include the use of a range of qualitative methods to explore a very private topic with people who may never have spoken about menstruation to another person. Data triangulation was applied to compare information generated through different modes, and data saturation was perceived to have been reached. Another strength was the inclusion of people with disabilities on the research team; we believe they were able to challenge misconceptions of carers that people with disabilities are unable to work and therefore always reliant on family members.

In terms of limitations, several possible sources of bias arose due to different types of missing data. Though participants were recruited from each impairment group (communication is included in ‘multiple’), we were unable directly interview one person with an intellectual impairment. As participants in this functional domain were unable to fully understand the consent process, their carers were interviewed instead, which may not reflect their own perspectives.

The lead author’s employment history includes working as a WASH practitioner focused on mainstreaming disability inclusion in development, which influenced the data collection tools applied and topics explored. For instance, experience and studies conducted dictate that people with disabilities faced more physical barriers to accessing WASH facilities than people without disabilities, so participants were observed using them and asked about barriers faced during interviews [27, 70, 71]. To minimise potential bias, a systematic review of relevant literature was conducted before the study to understand existing evidence and question personal assumptions about the barriers faced and data collection tools to apply [5].

AS and AG have a disability and we were cognisant that their experiences may introduce bias. To manage this, the week-long training for the research team included encouraging the team to mainly talk about their own experiences with participants after the interview. We also met regularly to discuss potential bias in data collection and analysis of results.

## Conclusion

This study highlights the additional barriers to MHM that people with disabilities, and their carers experience, as well as the negative impacts that these have on their physical, emotional, mental and social wellbeing. Issues related to MHM for people with disabilities is even more complex than for others in the population, due to the additional disability discrimination and constraints experienced, so require innovative and adapted solutions to existing MHM approaches that often fail to reach them. Even though MHM is not explicitly included in the Sustainable Development Goals (SDGs), it is essential for achieving the goals on gender, health and education [72]. Disability is the largest minority group, so attention to, and resourcing for disability inclusive MHM must be prioritised for progress to be made within the last nine years of the SDGs, which aims to ‘Leave No One Behind’.

## Supplementary Information

**Additional file 1.** In depth interview question guide, product attribute assessment (market survey) and accessibility and safety audit for carers.

**Additional file 2.** In depth interview question guide, product attribute assessment (market survey) and accessibility and safety audit for people with disabilities.

**Additional file 3.** PhotoVoice – guidance for researchers, which explains the purpose of PhotoVoice, how to identify participants, consent process, and a step by step guide on how to conduct PhotoVoice and ranking with participants.

**Additional file 4.** PhotoVoice images taken by participants (Figs. 1, 2, 3, 4, 5, 6, 7, 8, 9, 10 and 11), ranked according to perceived level of importance. The images relate to the following results: ‘The barriers to MHM differ depending on the person’s impairment’, ‘Disposable menstrual pads are preferable, but disposable practice and service are inadequate’, and ‘Impacts of menstrual restrictions’.

**Additional file 5.** Menstrual product preference across impairment type, containing Table 1: Most preferred product according to the person’s impairment, and Table 2. Least preferred product according to the person’s impairment.

## Abbreviations

CIUD: Centre for Integrated Urban Development
CRPD: Convention on the Rights of Persons with Disabilities
KIRDAC: Karnali Integrated Rural Development and Research Centre
LMICs: Low- and middle-income countries
LSHTM: London School of Hygiene and Tropical Medicine
MHM: Menstrual hygiene management
WASH: Water, sanitation and hygiene
WEDC: Water and Engineering Development Centre
