# Supplementary material for: Qualitative study exploring the barriers to menstrual hygiene management faced by adolescents and young people with a disability, and their carers in the Kavrepalanchok district, Nepal
Source: BMC Public Health. 2021 Mar 10;21:476. doi: 10.1186/s12889-021-10439-y (PMC7944905; doi:10.1186/s12889-021-10439-y)
Supplement: Supplementary file 1 — Additional file 1. In depth interview question guide, product attribute assessment (market survey) and accessibility and safety audit for carers. [file 12889_2021_10439_MOESM1_ESM.pdf]

In Depth Interview Question Guide for Carers

Materials and supplies for in depth interview

- One digital recording device
- Spare Battery for recording device
- Notebook for note-taking and pens
- Camera
- Refreshments

Use of these questions

These questions are a guide only. In practice, field staff should familiarise themselves with these questions and adapt them to suit the participant and their responses. This responsiveness is important to ensure that the participant feels at ease and able to talk about these topics. The interview should feel more like a conversation rather than a question and answer session. It is important to establish a rapport with participants, so begin with some general questions about their family and their interests before delving into what are quite private topics relating to menstrual hygiene management. Questions should be added where a participant mentions something of interest or touches on something that needs further clarification. Questions can be skipped where a participant answers the question in a previous response. You may not need to go through all questions.

Risks

If there is a concern that the Carer does not understand what informed consent entails, do not interview him / her.

To be completed for each interview

Name of Interviewer	FIRST NAME	SURNAME	
Name of note taker	FIRST NAME	SURNAME	
District	Village / Metropolitan area / Municipality	Household number	
Name of the parent / carer	FIRST NAME	SURNAME	
Age			
Caste			
What is your main source of income?	*Circle only one answer*<br>1. Agriculture / livestock<br>2. Trader (food / non-food items)<br>3. Craftsperson<br>4. Small business / shop owner<br>5. Manual labourer<br>6. Other (specify)		
Name of Primary Participant	FIRST NAME	SURNAME	
Has written informed consent been obtained from the primary participant?	Yes	No	N/A
If the parent / carer has a communication / intellectual disability, have they been given the choice about who they want to be interviewed with if they prefer not to be interviewed by themselves?	Yes	No	N/A

Date of interview		Start time:	End time:
Interview number		Audio interview recording number:	
Additional information			

INSTRUCTIONS: INTERVIEWER TURN ON THE TAPE RECORDER AND SAY CLEARLY THE DATE, TIME, LOCATION, AND THE INTERVIEWER'S NAME

Opening/ rapport building questions

- Who do you live with?
- Who do you care for?
- Do you go to school / work? Tell me about that.

KEY QUESTIONS

Now we are going to move on to our discussion of more specific questions about menstruation.

1. **Could you tell me a bit about the disability that [primary participant's name] has?**

2. **How long have you been caring for [primary participant's name]?**

3. **What support do you give [primary participant's name]?**

    - What are the main tasks?
    - Are any of these related to self-care (i.e. toileting, washing her body, washing her clothes and bedding, fetching water for her)?

4. **Does [primary participant's name] ever have difficulty controlling when they urinate or defecate?**

    - How do you deal with that?
    - What are the challenges (i.e. getting her to the toilet, washing her, cleaning her clothes / bedding, taking her out of the house)?
    - Do you use products (i.e. cloth, pad, nappy)? What are these? Where do you get them?

5. **Can you tell me about the first time [primary participant's name] got her period?**

    Context:
    - Where was [primary participant's name]?
    - What did she do?
    - What did you do?
    - How did you help her manage it?

    Attitudes/emotions:
    - What was your immediate reaction or feeling?
    - How did [primary participant's name] react?
    - How did that make you feel?

    Knowledge:
    - Did you know how to help [primary participant's name] manage?

- Could you explain what was happening to [primary participant’s name]?
- What did you say?
- Did she understand?
- Did anyone tell you how to help [primary participant’s name] manage her period?
- Who? (Family, friends, teachers, health worker)?
- What did they tell you?
- Did anyone help you care for [primary participant’s name] when she had her period?

Resources/management/practices:
- What did you use (pads, cloth, tissues, etc.)?
- Who/where did you get materials from?
- Where did you go to change / wash her menstrual material (latrine, separate room, outside, etc.)?
- Do you remember if there was water, soap, privacy?
- Did you have any challenges using the place where you helped her wash / change her menstrual material? Please explain?
- Where did you go to help her wash her body?
- How did you feel about helping [primary participant’s name] manage their first period?

Support:
- Did you tell anyone when [primary participant’s name] got their period for the first time? Please explain.
- If yes, who did you talk with? Why?
- What did you talk about?
- Did you try to hide it or keep it a secret? Why?
- Who did you not want to know? Why?

6. **Can you tell me about the most recent time [primary participant’s name] got her period?**

Context:
- Where were you both?
- Was [primary participant’s name] prepared for it? How?
- Were you prepared for it? How were you prepared?

Attitudes/emotions:
- How did you feel about helping [primary participant’s name] manage their period (proud, pleased, scared, worried, frustrated, no negative or positive feelings at all)? Why?
- Has this changed since [primary participant’s name] first period? Why?

Knowledge:
- Did you know how to help [primary participant’s name] manage?
- Could you explain what was happening to [primary participant’s name]?
- What did you say?

- Did she understand?
- Do you have more information now about how to help [primary participant’s name] manage her period?
- What information is that? Who gave that to you information?
- Did anyone help you care for [primary participant’s name] when she had her period?

Resources/management/practices:

Instructions: you may want to complete the Product Attribute Assessment now and run through the following probing questions, or you can complete the Product Attribute Assessment at the end of the IDI questions.

- What material / product did you use (pads, cloth)?
- Does [primary participant’s name] normally use those?
- Do you use different supplies for different days of [primary participant’s name] period (light or heavy menstrual flow)?
- Is it difficult for the [primary participant’s name] to use them? Why?
- Do you use different materials / products to manage your own period? Why?
- Who/where did you get [primary participant’s name’s] menstrual material / product from?
- How often do you change them?
- Where do you change them?
- Where do you go to wash her menstrual materials (if not disposable)?
- Where do you dry her re-usable materials? (Inside? Outside? In sun? In shade?)
- Where do you store her materials when you do not have your period?
- Where would you dispose of her menstrual materials (if not re-usable)? Is this method of disposal OK or would you prefer other options?
- Where did you and [primary participant’s name] go to wash her body?
- Does [primary participant’s name] disability make it hard to wash her body?
- Do you feel safe in the place you clean her?
- Do you have more washing to do when [primary participant’s name] has her period (i.e. clothes, bedding)?
- If you do, how many more times do you do this than when [primary participant’s name] does not have her period?
- Does [primary participant’s name] have pain? How do you help her manage the pain (e.g. massage, warm cloth, paracetamol)?
- What’s your biggest worry or challenge when [primary participant’s name] has her period (i.e. leaking, heavy bleeding, availability of water for washing)?

Support:

- Does anyone ever provide materials or supplies for [primary participant’s name]? Who?
- If someone provides materials for [primary participant’s name], where do they get the materials? Do they cost something?

- Who did you not want to know that [primary participant's name] had her period? Why?

7. **Has anyone ever spoken to you about lightening [primary participant's name's] menstrual flow?**

If yes:
- Explore if this is sterilization or lightening the menstrual flow.
- Why have you considered that?
- How did you hear about that?
- Who did you speak to about it?
- What did they tell you?
- What did you do?
- Did [primary participant's name] understand what was happening? Did they agree to it? Can you tell me about that?
- What difference has it made to you and [primary participant's name]? How do you both feel about that?

If no, move to the next question.

8. **Has anyone ever spoken to you about lightening [primary participant name's] menstrual flow? Can you tell me about that?**

- Who spoke to you?
- What did they tell you?
- What options did they give you?
- What happened? Explore reasons for lightening the menstrual flow; who made the decision; if the primary participant knew what was happening; did she consent?
- What difference has it made to you and [primary participant's name]? How do you both feel about that?

If no, move to the next question.

9. **Are there any activities you don't want [primary participant's name] doing when she has her period? Why?**

- How does [primary participant's name] feel about that?

10. **Are these restrictions different for her to females who do not have a disability? How?**

11. **Looking back at your experiences, what have you learned about how to help [primary participant's name] manage her period?**

Management/practices:
- Do you know when [primary participant's name's] period is coming? How?
- How do you normally prepare for her period?

12. **Do you know when [primary participant name's] period is coming?**

   - How does that help you prepare and manage her period?

13. **What would help you to better support [primary participant's name] to manage her period? (i.e. information, products, a private place to wash her body, her menstrual material and dry it)?**

14. **Of the information you received (if any), what has been the most useful? Why was it useful?**

   - Who gave you that information?
   - Of the support you have received (if any), what has been the most useful? Why?
   - Who provided that support?
   - How would you like to receive information about how you can help [primary participant's name] manage her period (radio, television, newspaper, healthcare centre)? Why?
   - Who would you like to receive that information from (healthcare worker, friend, family member)? Why?

15. **Are there any advantages to caring for [primary participant's name] when they have their period (ie knowledge that she has a good menstrual cycle)? Please explain.**

16. **If you had a relative or friend whose daughter has a disability, what advice would you give her before her daughter starts menstruating? What would you say?**

17. **We are going to come up with recommendations for organisations and healthcare professionals that could help improve menstrual hygiene management for people with a disability. What advice would you like us to pass along to them that you think would make things better for you when [primary participant's name] has her period?**

Product Attribute Assessment

I would like to see menstrual products you use. Please can you set out all the menstrual hygiene products you use?

1. Ask about each item displayed:
    a. What are its good qualities (probe into physical accessibility, acceptability, affordability, appropriateness and quality)?
    b. What are its less good qualities (probe into physical accessibility, acceptability, affordability, appropriateness and quality)?
    c. How long has this product been used?
    d. Have you tried using any other product?

2. Introduce other products that are available on the local market (obtained through the market survey). Ask the participant if they have ever used any of the products? *State what these are into the audio voice recorder.*

3. Ask participants what they feel about these products. *State which product are being discussed into the audio voice recorder.*
    a. Would you be willing to pay the price of this? Show the costs of each product (displayed on the bottom of the packaging)?
    b. What would your personal preference be? Why? (Probe into physical accessibility, acceptability, affordability, appropriateness and quality)?
    c. How would other people perceive these products?
    d. Could you use, wash and dispose of these products independently and privately? If not, why not?

4. Give the shop bought products and the products currently used to the participant. *State what these are into the audio voice recorder.* Ask the participant to:
    a. Put all the products in a line
    b. Reorder the products in preference order. Left being the least preferred option. Right being the most preferred option
    c. Ask the participant why they have ordered them in that way
    d. *Take a photo of the product line and state the order into the audio voice recorder.*

Accessibility and safety audit

<table>
<tr><td colspan="2">Ask the user to show you where she manages her menstruation / where the Carer manages the menstruation of the person s/he is caring for.</td></tr>
<tr><td>Type of facility (i.e. latrine, wash room, behind a bush/tree):</td><td></td></tr>
<tr><td>Location:</td><td></td></tr>
<tr><td>General description of the facility:</td><td></td></tr>
<tr><td colspan="2">Ask the user to into and show how they can/cannot use the facility. Make a note of any features that make it difficult to use.</td></tr>
<tr><td>Getting there (observe):</td><td></td></tr>
<tr><td colspan="2">Checklist
• Distance from house to facility.
• Is the path wide enough for the primary participant?
• Is the path level and firm, with nothing to trip up?
• Is the path surface slippery when either dry or wet?
• Are there obstacles that block the path, or make it easy to trip especially for visually impaired people?
• Are slopes too steep?
• If used at night, is the path lit?
• Are there any parts of the path which make people feel unsafe when using it? If so why?</td></tr>
<tr><td>Getting in/on/out (observe):</td><td></td></tr>
</table>

Checklist<br><u>Steps:</u><br>• Are they even or uneven, firm or broken, non-slip or slippery?<br>• Is there a handrail for support?<br><u>Entrance:</u><br>• Is it wide enough for the user to enter?<br>• Is the door easy to open?<br>• Is the door easy to lock and unlock?<br>• If someone faced harassment or other safety risks when using the facility would they be able to safely get away from the facility?	
Safety of use (ask the participant)	
Checklist<br>• Do you feel safe when using the facility?<br>• Are there any particular times of day or night when you feel less safe?<br>• Is there any way that anyone can easily see inside the facility?	
Inside (observe)	

<table>
<tr><td colspan="2">Checklist
• Is there enough space inside for a participant and her Carer (if she needs support) to move around easily?
• When the door is closed is there enough light to see?
• Do the roof / window (if applicable) provide adequate privacy for user/s?</td></tr>
<tr><td>Water (observe and ask the participant)</td><td></td></tr>
<tr><td colspan="2">Checklist
• Is there an internal water point? Describe.
• Can the water be used to wash the body and the menstrual cloth?
• Is soap or ash available?
• Can the water and soap be easily reached by all users?</td></tr>
<tr><td>Disposal: Is there a place where washed reusable menstrual cloths be dried, or where it can be thrown away (observe / ask the participant)?</td><td></td></tr>
<tr><td colspan="2">Checklist
• Can the cloth be hung up?
• Is it in sunlight to dry the cloth?
• Is there a bin with a lid for sanitary disposal?
• Is there a safe location for the final disposal of the sanitary waste (i.e. incinerator, a place to bury it or disposal into municipal waste collection systems)?</td></tr>
<tr><td>Any additional information or comments</td><td></td></tr>
</table>

Annex 1: Algorithm outlining referral options offered or actions taken in response to disclosures of violence

Referral Type	Age	Discloses	Defined as:	Primary course of action/person notified	Time frame for action	Finish to interview
Mandatory	Any age	Use of severe physical or sexual violence against a child	Burning, choking, stabbing, whipping, beating up, or any similar act; physically forcing or coerced sex	Research supervisor notifies project manager same day. Project manager notifies <<named individual>> the same day.	Discussion of each case and a plan for any further action within one day of receipt of the case.	[INTERVIEWER]: "Because of some of what you've told me, I am concerned that a child in your care may be at risk and I have a professional responsibility to let somebody else know. What I'm going to do is talk to my research team supervisor and they will pass your details to <<named individual>>. I am not going to pass anything you said to me in this room to anybody else. Is there anything you'd like to ask me?"
Optional	Age over 18 years	Forced sexual intercourse within the past week, or obvious untreated physical injuries	In **past week**: threatened or pressured into sex; physically forced sexual intercourse or doing sexual things; suffered cuts, loss of consciousness; dislocated, sprained, fractured or broken bones; untreated injuries or severe injuries (requiring medical attention) reported as a result of physical or sexual violence; signs of acute malnutrition /neglect	With consent of participant: research team supervisor telephones <<named individual>> immediately from the field. Immediate transport to health centre is arranged by <organisation> in consultation with research team supervisor; concurrent arrangements made for other support as necessary by <organisation>, other adults notified at request of child. Transport arrangements may involve the research team, 3rd parties, or staff of <organisation> depending on the time frame and particulars of the case.	Participant taken to health centre immediately/same day/next day depending on the particulars of the case.	[INTERVIEWER]: "Because of what you've told me today, I am concerned about your health and safety. What this means is that if you want me to, I will to arrange to bring you to the health centre, to make sure that you are OK. If you agree, what will happen next is that I will talk to my friend <<named individual>>, who works at <organisation> in child protection. She will arrange for you to visit the local health centre, and will want to speak with you. It's part of <<named individual>>'s job to help make sure that young people in your community are safe and taken care of. She/he is very experienced and has helped many young people. You can ask me what is going on and what is going to happen

						next at any time. How do you feel about that?<br><br>Are there any other adults you would like me to help you get in touch with about the things you have told me? Would you like me to inform your parents/caregivers? It is your choice.<br><br>What is the best way for <<named individual>> to get in touch with you about this, for example, at school, or at home, or some other way?”
Mandatory	Under age 18 years	Forced sexual intercourse within the past week, or obvious untreated physical injuries	In **past week**: threatened or pressured into sex; physically forced sexual intercourse or doing sexual things; suffered cuts, loss of consciousness; dislocated, sprained, fractured or broken bones; untreated injuries or severe injuries (requiring medical attention) reported as a result of physical or sexual violence; signs of acute malnutrition /neglect	With or without consent of participant: research team supervisor telephones <<named individual>> immediately from the field. Immediate transport to health centre is arranged by <organisation> in consultation with research team supervisor; concurrent arrangements made for other support as necessary by <organisation>, other adults notified at request of child. Transport arrangements may involve the research team, 3rd parties, or staff of <organisation> depending on	Participant taken to health centre immediately/same day/next day depending on the particulars of the case.	[INTERVIEWER]: “Because of what you’ve told me today, I am concerned about your health and safety. What this means is that I will to arrange to bring you to the health centre, to make sure that you are OK. What will happen next is that I will talk to my friend <<named individual>>, who works at <organisation> in child protection. She/he will arrange for you to visit the local health centre, and will want to speak with you. It part of <<named individual>>’s job to help make sure that young people in your community are safe and taken care of. She is very experienced and has helped many young people. You can ask me what is going on and what is going to happen next at any time.

				the time frame and particulars of the case.		Are there any other adults you would like me to help you get in touch with about the things you have told me? Would you like me to inform your parents/caregivers? It is your choice.<br><br>What is the best way for <<named individual>> to get in touch with you about this, for example, at school, or at home, or some other way?”
Optional	Any age	All other disclosures causing concern	For example, other less severe forms of violence, mental health issues	Researcher offers to contact <organisation>; <<named individual>> will then provide list of agencies which may be able to provide support, and facilitates contact according to wishes of the participant	Discussion of each case and documentation of further action within three weeks of receipt of the case.	“Thank you for sharing those things with me. If you want, I can recommend somebody who might be able to help you. If you say yes, I would like to put you in touch with my friend <<named individual>>, who works at <organisation>. She/he may be able to help put you in touch with other organisations that can assist you. Do you think you would like me to contact <<named individual>> for you?<br><br><<if participant says yes>><br>“What is the best way for <<named individual>> to get in touch with you?”
