# Supplementary material for: Qualitative study exploring the barriers to menstrual hygiene management faced by adolescents and young people with a disability, and their carers in the Kavrepalanchok district, Nepal
Source: BMC Public Health. 2021 Mar 10;21:476. doi: 10.1186/s12889-021-10439-y (PMC7944905; doi:10.1186/s12889-021-10439-y)
Supplement: Supplementary file 3 — Additional file 3. PhotoVoice – guidance for researchers, which explains the purpose of PhotoVoice, how to identify participants, consent process, and a step by step guide on how to conduct PhotoVoice and ranking with participants. [file 12889_2021_10439_MOESM3_ESM.pdf]

PhotoVoice – guidance for researchers

Purpose of PhotoVoice

This exercise is designed to investigate the barriers to menstrual hygiene management that the primary participants face and how these barriers are prioritised (if at all) by the primary participants. This method aims to empower participants with photography skills so that they are able to share their personal perspectives and experiences of how these challenges affect the lives of individuals with disability. This method is particularly important for the exploration of this topic which tends to be difficult to talk about since it is very private.

Requirements

- Digital camera, SD card and spare battery
- Photo printing facilities
- Voice recorder
- Notebook and pen

Identifying participants

It may not be possible to do PhotoVoice with all individuals with disability. For this exercise it is important that participants are able to follow instructions and think critically about the task being given. Because of the nature of digital cameras motor skills are important. For individuals with severe intellectual impairments this task is likely to be difficult. However since it is also a very practical interactive exercise, it may be possible to undertake the activity with those who have mild intellectual impairments. Experience indicates that not only does this task work well with those who have physical disabilities, but it can also be used effectively with individuals who have sensory impairments (including those who are totally blind) and could work well with those who have hand/arm impairments.

Consent and explanation of the process

The consent process must be done very thoroughly to ensure that the primary participant understand the purpose of the activity and what they are agreeing to. Each primary participant will have been asked for their consent to be given through the in-depth interviews.

Consent to take part in the research must be signified in writing by the primary participant wherever possible. Where the participant is unable to give full consent or is 15 years old, a guardian/career must also provide written consent. Where primary participants or their caregivers are illiterate they should use a thumbprint to signify their consent.

A secondary process of obtaining permission should be done after the photos have been taken. This process relates to how the photos can be used and how they should be acknowledged. This is done after the process so that the participant can make a better judgement about how they wish them to be used.

PhotoVoice Process:

The PhotoVoice process used in this research was partly taken from the process developed by photovoice.org (http://www.photovoice.org/methodologyseries/inclusivemethodology/designing.htm)

1. **Understanding the camera** - A photographic camera (digital) will be provided to the participant. The field worker should explain simply how the camera works and allow the participant to observe (or feel if they are visually impaired) where the lens is and how the shutter works (a visually impaired person can listen to the shutter noise and feel the lens while closed, so not to leave marks on the lens). The field worker should take time to teach the participant the basic features of the device including how to switch the camera off and on, how to take a photo and how to view the photo. Allow the participant to have a go taking a photo to see what it feels like. It is important that while the participant is learning about the camera they are also holding it so that they begin to be familiar with how it feels. This is particularly important for people with sensory impairments so that they begin to learn how the buttons feel and how they are positioned. A useful starting point is to remind the individual to always use the wrist strap/head strap.

2. **Understanding photography** - Since many of your participants may never have seen a camera before and may have seen relatively few photos in their life, it is also important to explain the purpose of photography. We found that a simple way to explain this is to say that photography can serve several purposes. You can use photography to capture a moment you want to remember as if it was real again. Give an example of this by getting the participant to take a photo of the fieldworkers or their house, view the photo and point out that it looks exactly like it is in real life. Then explain that you can also use photography as if it was art by arranging things in a certain way that tells a story or creates a version of reality that can be explored or questioned. Give an example of this too – if you want to take a photo of the idea of 'hunger' it may be hard to show this literally. But you could use symbolism to show hunger. You might have a family seated in their living room, all looks normal except all of them have empty bowls in front of them. Explain that what we are going to do today is use photography to tell their story and creatively express their views.

3. **Understanding the elements of Photography** –
    a. **Landscape/Portrait -** Shooting can be done vertically (portrait) or horizontally (landscape). Show participants how this effects the image and explain that portraits can be better when you are focusing on a person and landscapes can be better when you want to capture more of the environment. For people who are visually impaired this can be demonstrated using a mount board window, which can be rotated and felt by the participants. A collection of tactile objects such as toys or fruit can be a good focus for this exercise – the window can be placed by the display in each position and the difference in what is contained in the 'photo' felt through the window.
    b. **Framing -** Explain that when taking a photograph it is not simply a matter of pointing towards the subject, but of deciding what is included in the photo – all or some of the subject, the subject and the background, the subject and what is above it etc. Tactile objects can be a useful reference for explaining this concept to someone who is visually impaired. Show the participant how to adjust the framing by using the zoom.
    c. **Foreground/background -** This must be explained in a verbal way as well as using their body as a reference. For example you can ask two participants to stand one in front of the other, and then explain who is in the foreground and who is in the background and what that would mean in a photograph (i.e. who would seem more important, more prominent, larger in the frame etc). Show the participant how to change the focus on the camera.

d. **Distance (only for people with visual impairments) -** When taking a photograph, it is very important to identify the distance to the subject, in order to be sure that it is framed as desired. This can be done by reaching with or laying out a cane, measuring it with steps, or measuring with the joints, such as hands, wrists, arms and forearms. It can be very reassuring for a photographer to know how a photo of a person will be framed if taken from the distance of one cane's length, for example.

e. **Focus/blur -** It is important to clearly identify the area that needs to be in focus. The photographer needs to remember that she can communicate different feelings or ideas depending on what is focused on in the photograph. Here is one way the concept can be explained in a way that makes sense to someone with no sight: When one touches a glass bottle, one identifies the material, its temperature, its dimensions and every detail that makes one recognize the object as a bottle. If this is done again with a thin cloth over the bottle, the details of the bottle won't be recognized so precisely. Nevertheless, one will still know it is a bottle, since some details, like its shape and size, are still recognised. This is what happens when one sees an image that's blurry or out of focus; one recognizes what it is but cannot make out the details clearly.

f. **Light -** Light plays an important role in a photograph since it produces different effects, which lead to different feelings in the observer. A person in darkness, for example, may convey an experience of feeling hidden, where as a person in bright light may convey confidence or nothing to hide. These effects need to be explained fully to blind or visually impaired photographers who will not necessarily realise the impact of the light on their work. To explain this try invite the participant to think about the warmth they feel on their face if they are in the sun and use this sensation to determine where the light is coming from. Also teach the participant how to use the flash setting for dark environments.

g. **Taking photos without showing a face** – explain how the participant can take portraits of herself and others without showing their faces. Techniques include:
   i. Taking photos with the light behind the subject so the figure will be in shadow.
   ii. Focusing on something behind or in front of the subject. This means that the subject will be in soft focus
   iii. Photographing a person's shadow
   iv. Taking a photo of someone from behind (the back of their head, their head / body etc)

4. **The photographic task** - Once the participant is comfortable with all this, set them their task. Explain that you would like them to take a total of 10 pictures. These pictures should represent their feelings about menstruation and how they manage it. Ask the participant if anything springs to mind. Help the participant make a list of these issues so that these things can be remembered. For each one, the participant should think about how she could represent the experience or feeling. If the participant needs more guidance, work through one example with her, but make sure she leads the process.
5. **Self-Directed portraits** - It is likely that in settings where participants are unfamiliar with cameras and photography that they will be keen to be in the photos rather than just taking them. If the participant wanted to be in the picture then they still had to direct the field researcher as to how they wanted the photo to look, providing direction on whether it was to be a portrait or landscape shot, what was in the foreground or background, how much of their body should be in shot etc.

6. Arrange a suitable time to return to the individual’s house to give them their photos and have a short discussion.

Printing and interview

When you return with the printed images ask the participant to caption each of the images. To explain what a caption is ask the participant to imagine that there is a person looking at the photo who doesn’t know them or anything about how they live – their task is to explain to them the experience they were trying to convey. Ask the participant to explain what story she was trying to tell when taking the photo.

Once they have done this get the photos and ask them to rank these from most important to least important. Document the order by taking a photo of them in order. Ask questions to explore why the participant has ordered the photographs in that way. Explore why the most important are particularly significant.
