# Supplementary material for: Qualitative study exploring the barriers to menstrual hygiene management faced by adolescents and young people with a disability, and their carers in the Kavrepalanchok district, Nepal
Source: BMC Public Health. 2021 Mar 10;21:476. doi: 10.1186/s12889-021-10439-y (PMC7944905; doi:10.1186/s12889-021-10439-y)
Supplement: Supplementary file 4 — Additional file 4. PhotoVoice images taken by participants (Figs. 1, 2, 3, 4, 5, 6, 7, 8, 9, 10 and 11), ranked according to perceived level of importance. The images relate to the following results: ‘The barriers to MHM differ depending on the person’s impairment’, ‘Disposable menstrual pads are preferable, but disposable practice and service are inadequate’, and ‘Impacts of menstrual restrictions’. [file 12889_2021_10439_MOESM4_ESM.pdf]

PhotoVoice images

Result: The barriers to MHM differ depending on the person's impairment

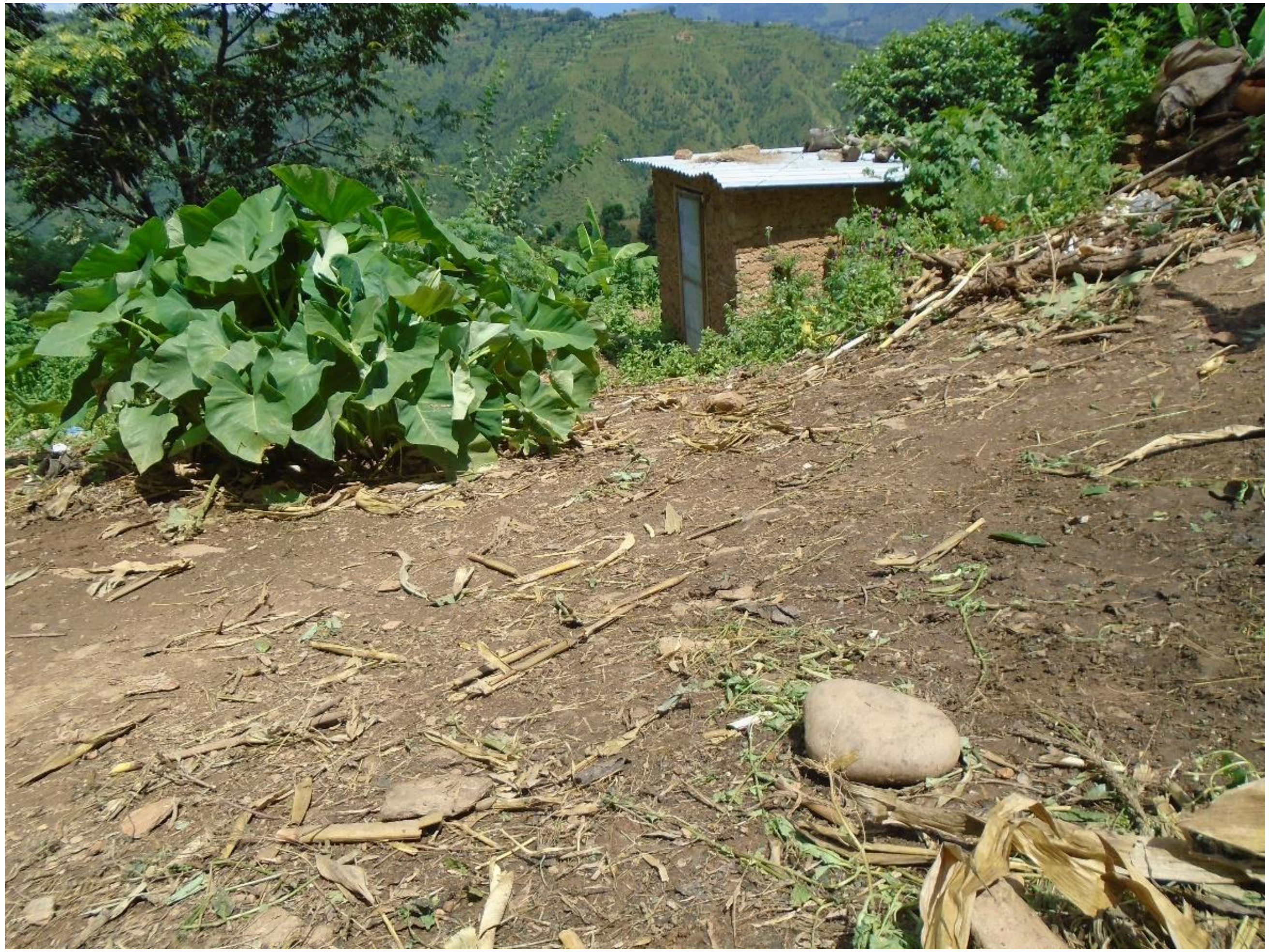

Fig. 1 “It is difficult to go to the toilet.” PhotoVoice image taken by Sharmila Tamang. Ranked 1 out of 4.

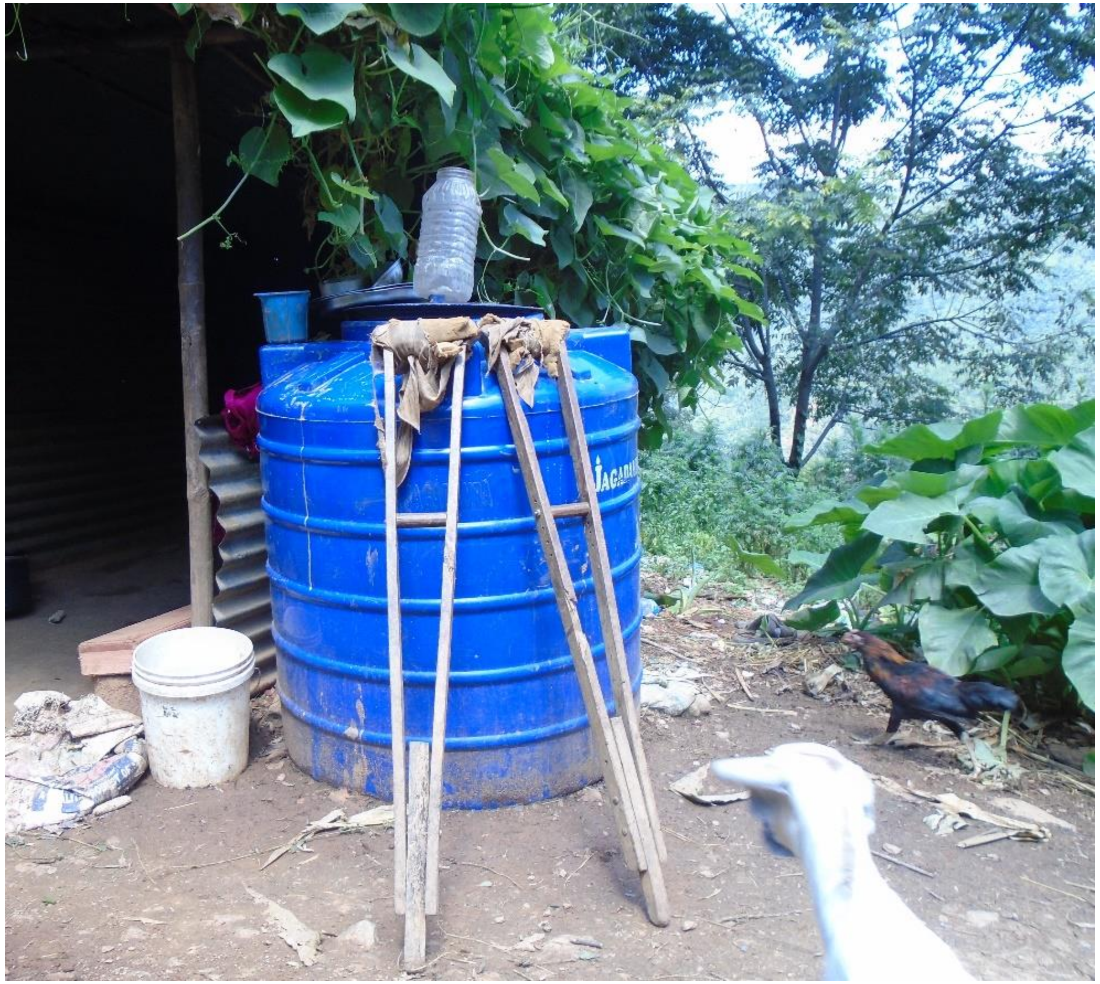

Fig. 2 “Water issue is also there, I would have to carry water which is difficult.” PhotoVoice image taken by Sharmila Tamang. Ranked 2 out of 4.

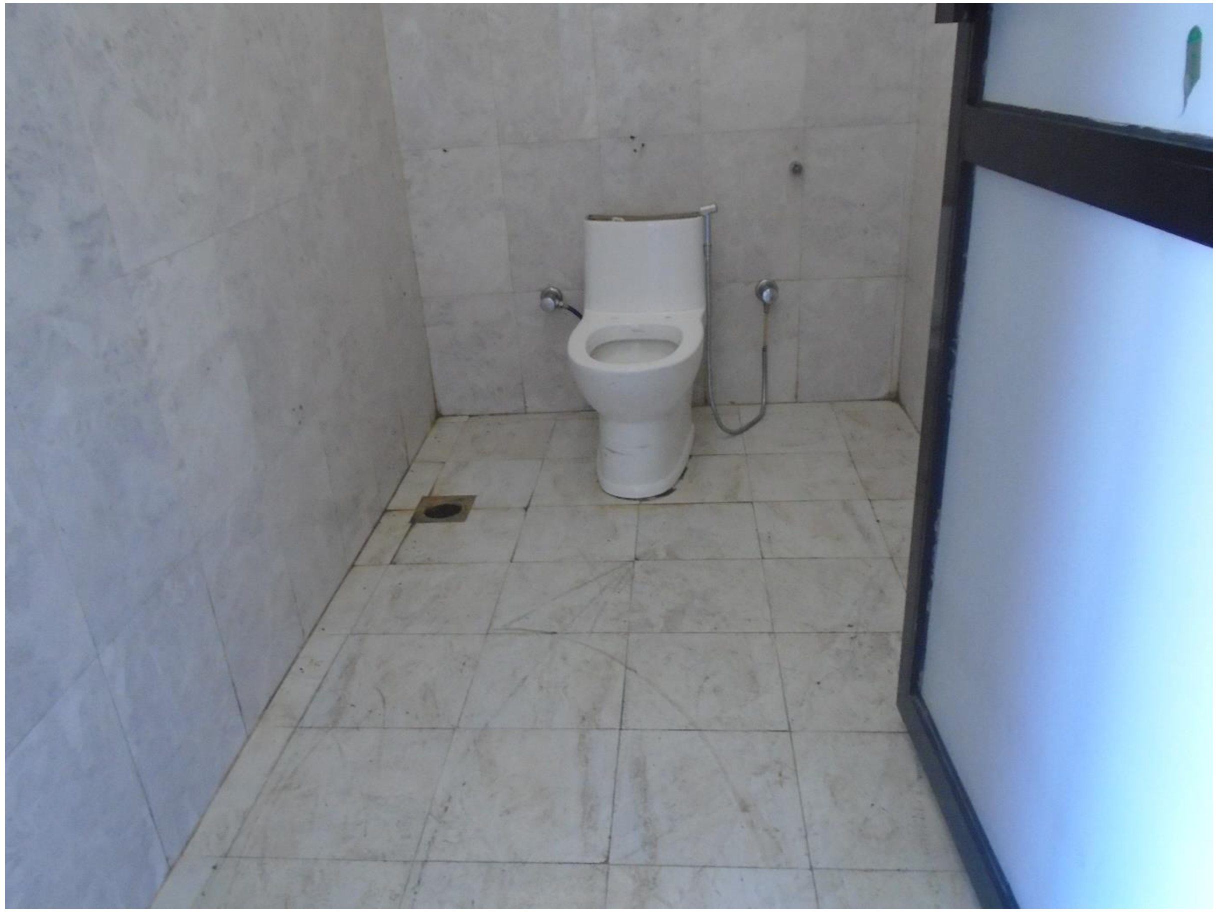

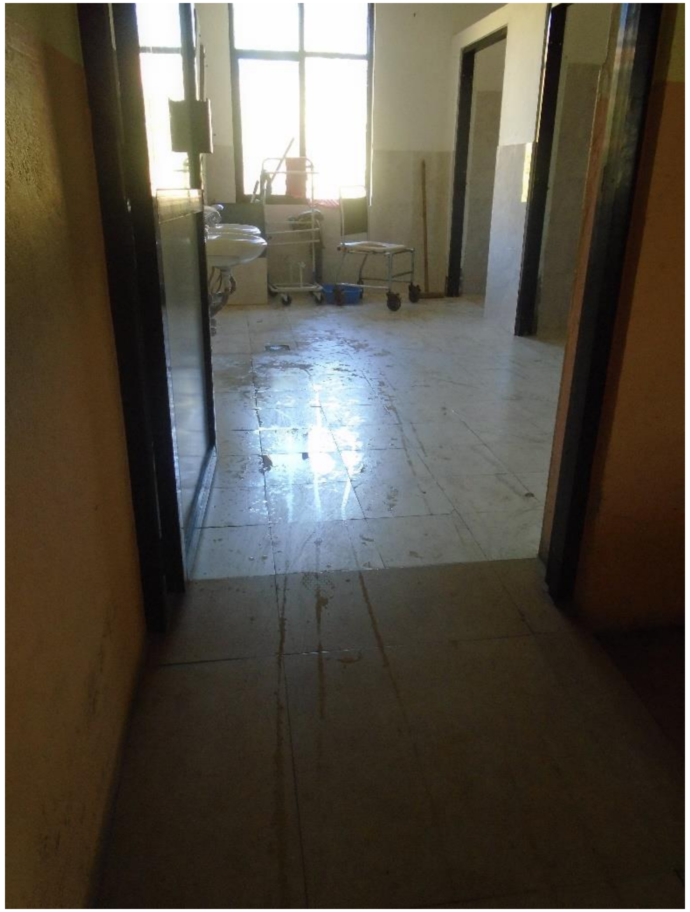

Fig 3. "It is not only easier to use this type of toilet for those with spinal cord injury but it is a necessity. So even in villages, these types of toilets should be built for people as not everyone can live in the cities. During period also it is difficult to stand to change pads so these kind is easier to use." PhotoVoice image taken by Babita Thapa. Ranked 1 out of 5.

Fig 4. "During period one should be careful using bathroom. Our legs might already feel weak (lulo) so we might fall down and meet an accident. Here the toilets are made for everyone to use but if it was to built at home for crutches user, it is to be made in a way that it is not slippery. Marble should not be used as it is slippery." PhotoVoice image taken by Babita Thapa. Ranked 3 out of 5

Fig 5. “For us to be able to wash our own clothes, bathroom should be made in such a way that we can wash our clothes ourselves while sitting on wheelchair. It will be much easier.

“I can’t wash clothes while standing or sitting. At home, I sit in a small stool but there is none here. If the washing space could be reached while sitting on a wheelchair, it would be good.” PhotoVoice image taken by Babita Thapa. Ranked 5 out of 5.

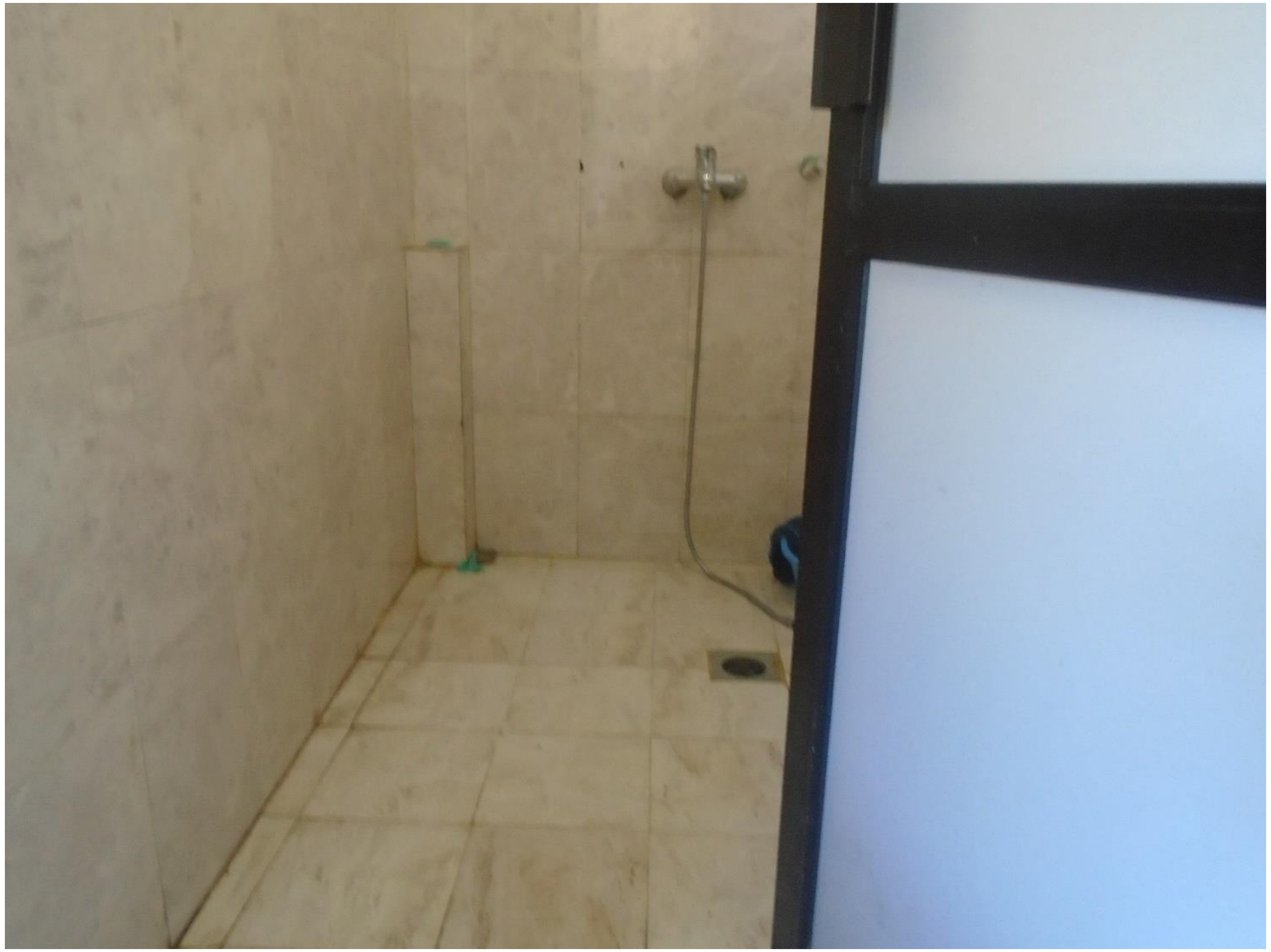

Fig. 6 “When I have to use the toilet, I need someone else to help with the latch otherwise I can’t do it myself.” PhotoVoice image taken by Tulasa Karki. Ranked 3 out of 4.

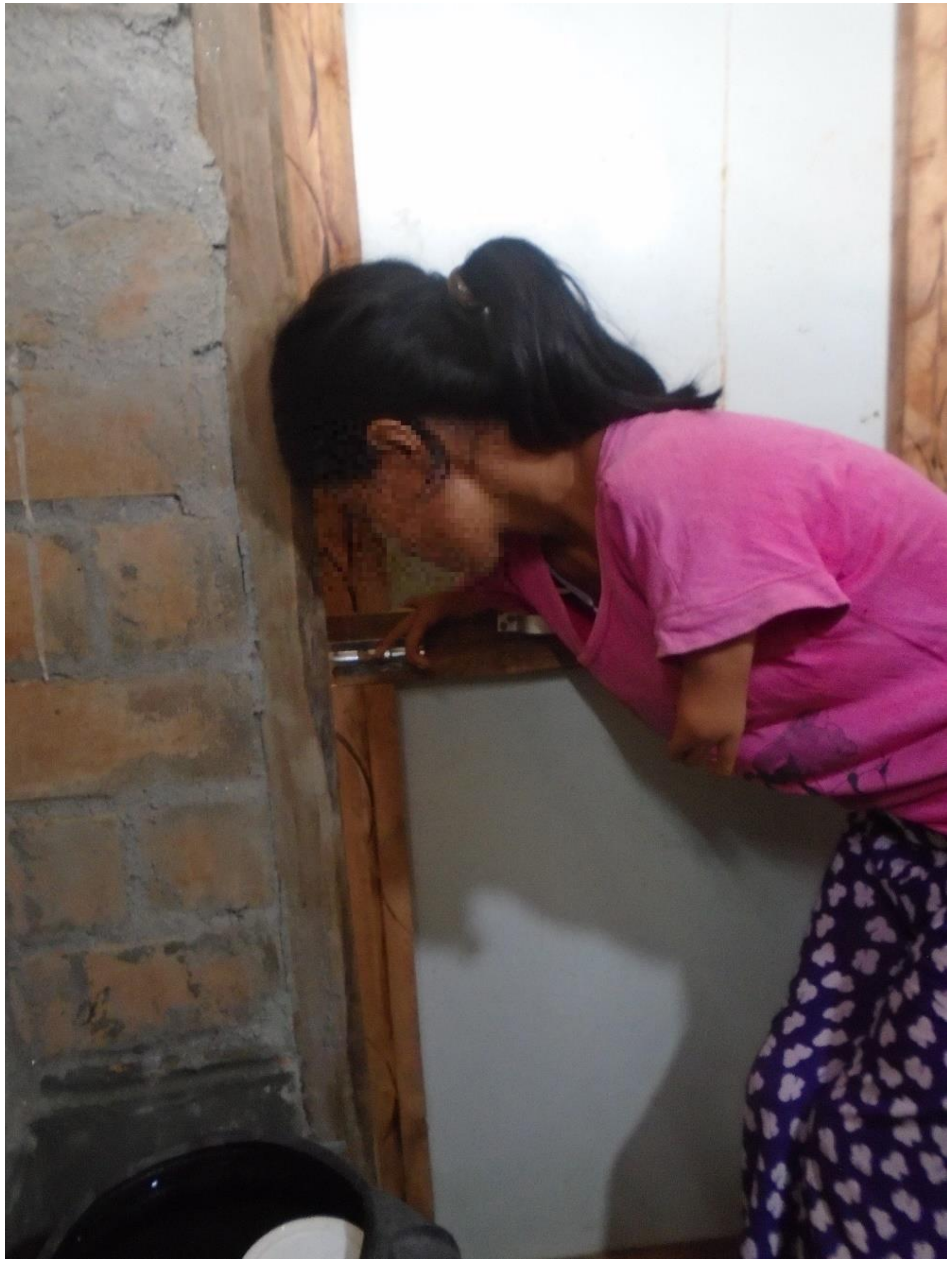

Result: Disposable menstrual pads are preferable, but disposal practices and services are inadequate

Fig. 7 “Pad is easy to use compared to cloth.” PhotoVoice image taken by Sharmila Tamang. Ranked 3 out of 4.

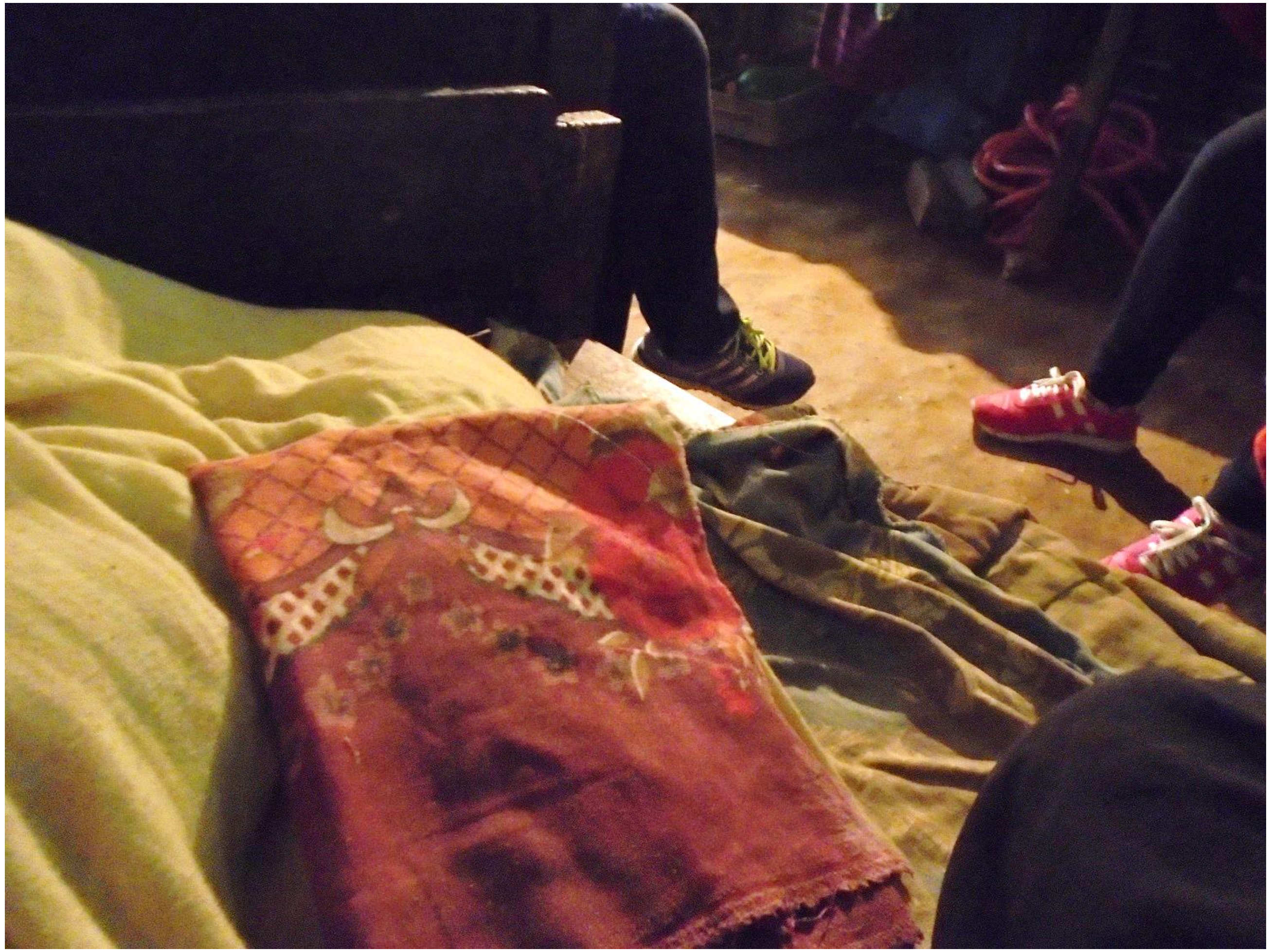

Result: Impacts of menstrual restrictions

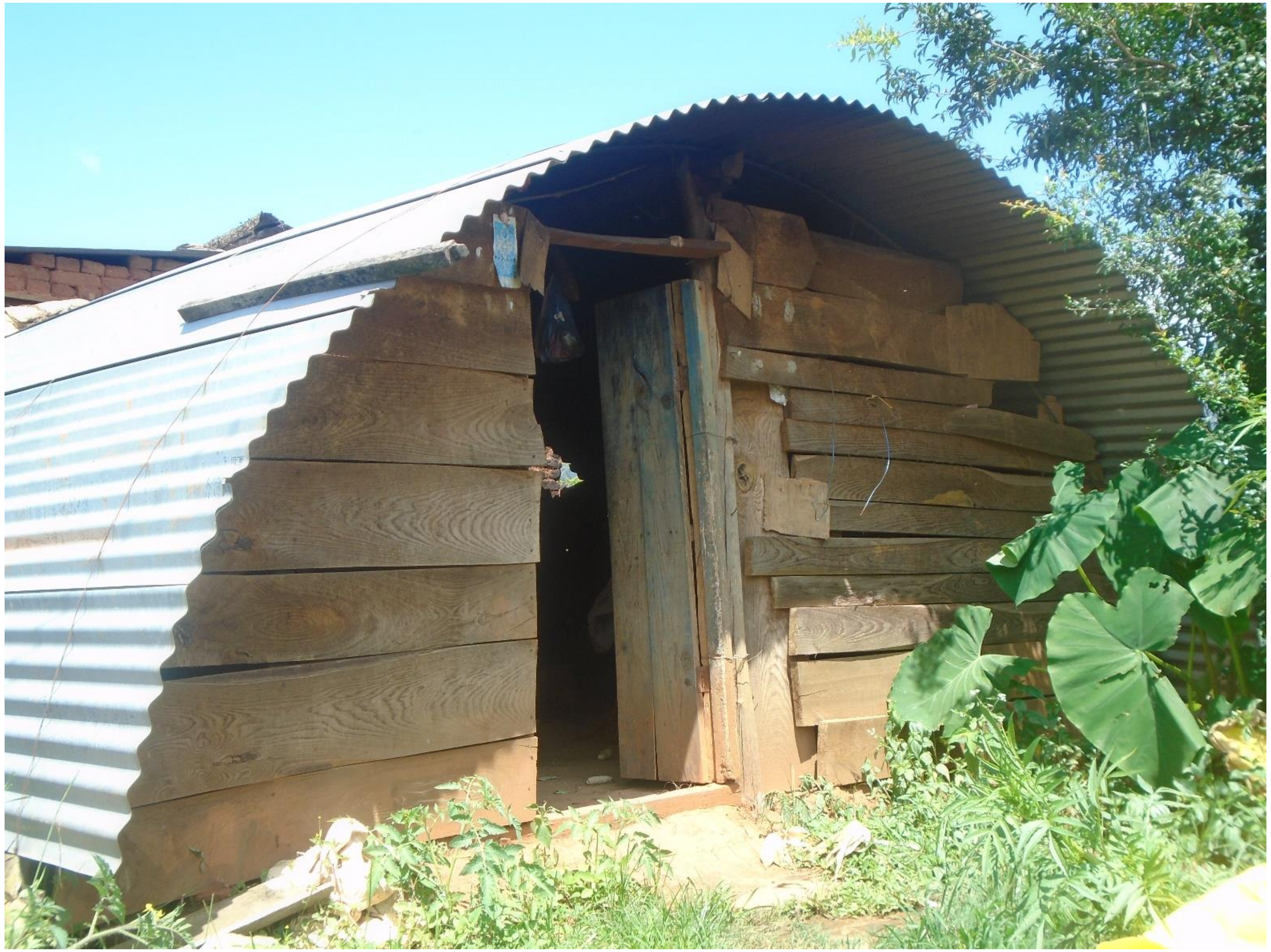

Fig. 8 "During menstruation we are not allowed to enter the house." The image is of the hut Tulasa sleeps in when menstruating. PhotoVoice image taken by Tulasa Karki. Ranked 2 out of 4.

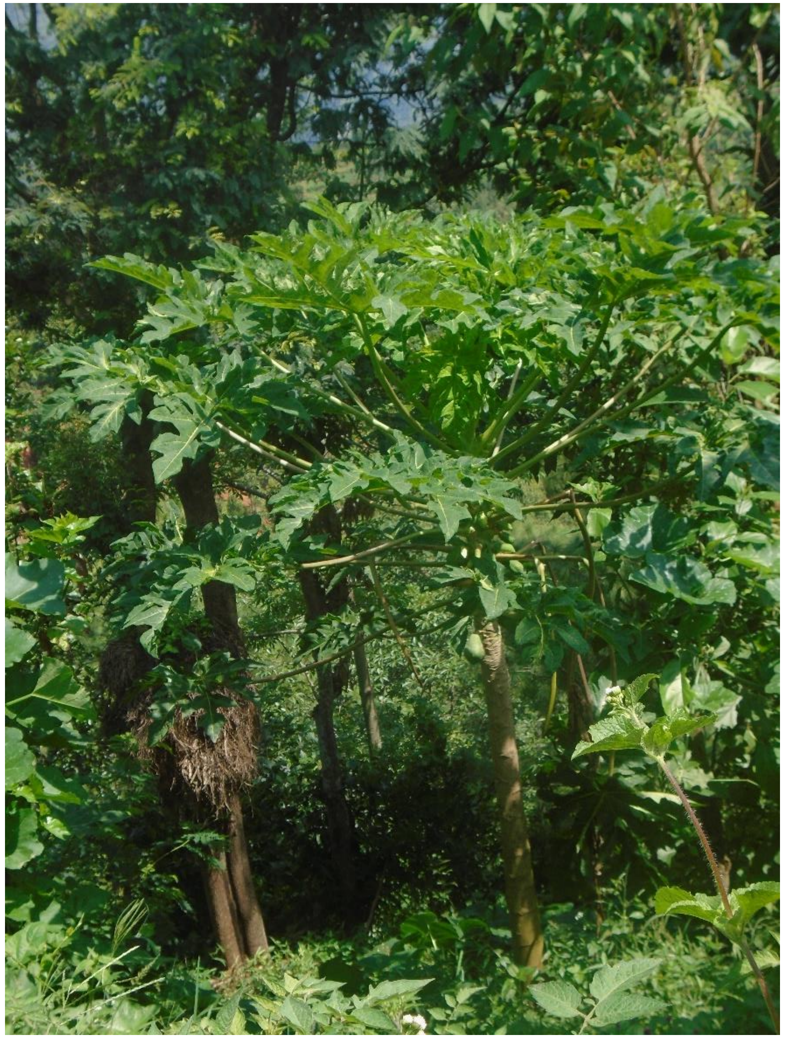

Fig. 9 "There is a belief that if you touch plants during period, it will rot." PhotoVoice image taken by Tulasa Karki. Ranked 4 out of 4.

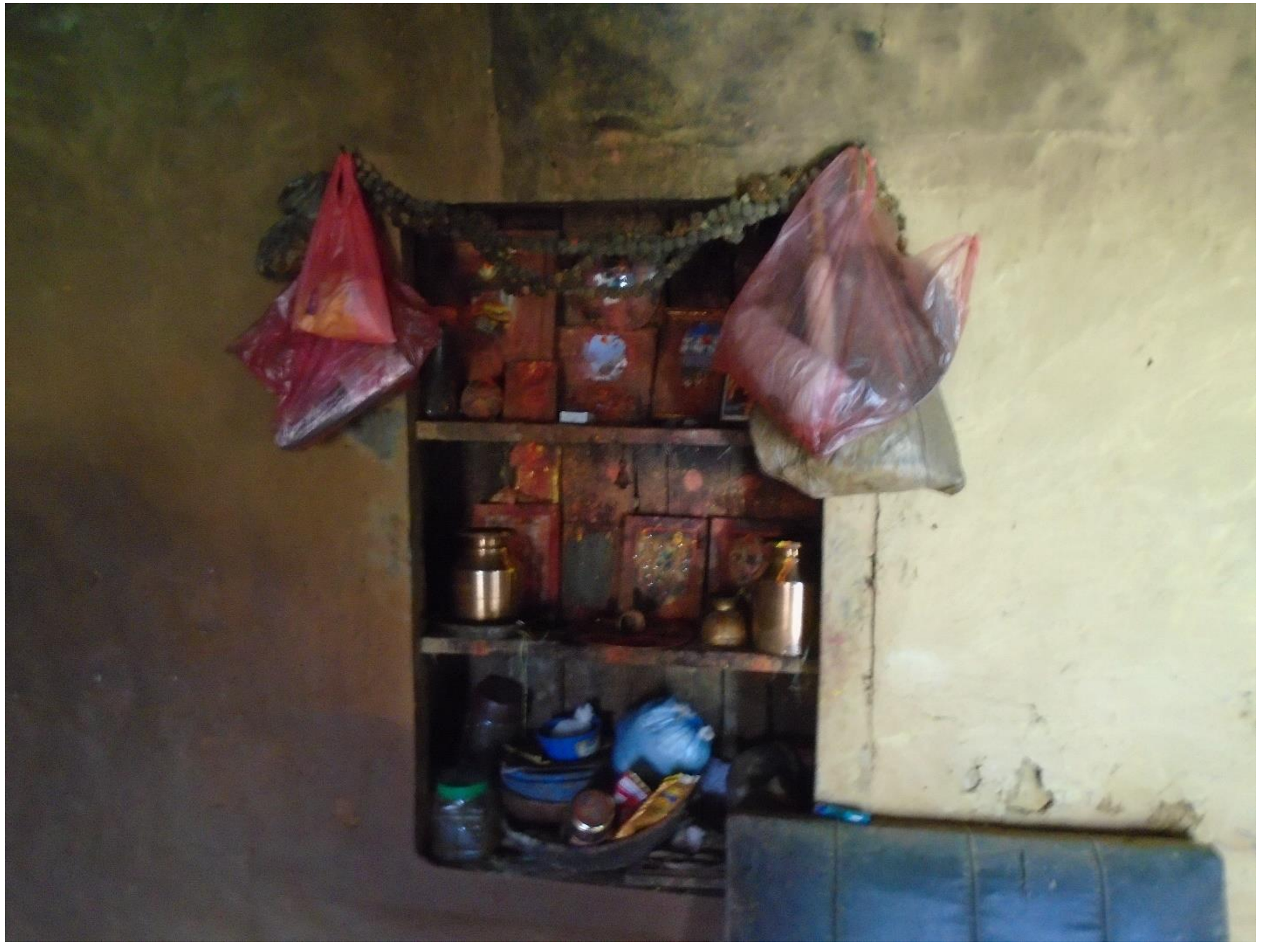

Fig. 10 "Cannot touch during period as per our tradition." The image is of worshiping area inside Bishnu's home. PhotoVoice image taken by Bishnu Maya Sapkota. Ranked 1 out of 4.

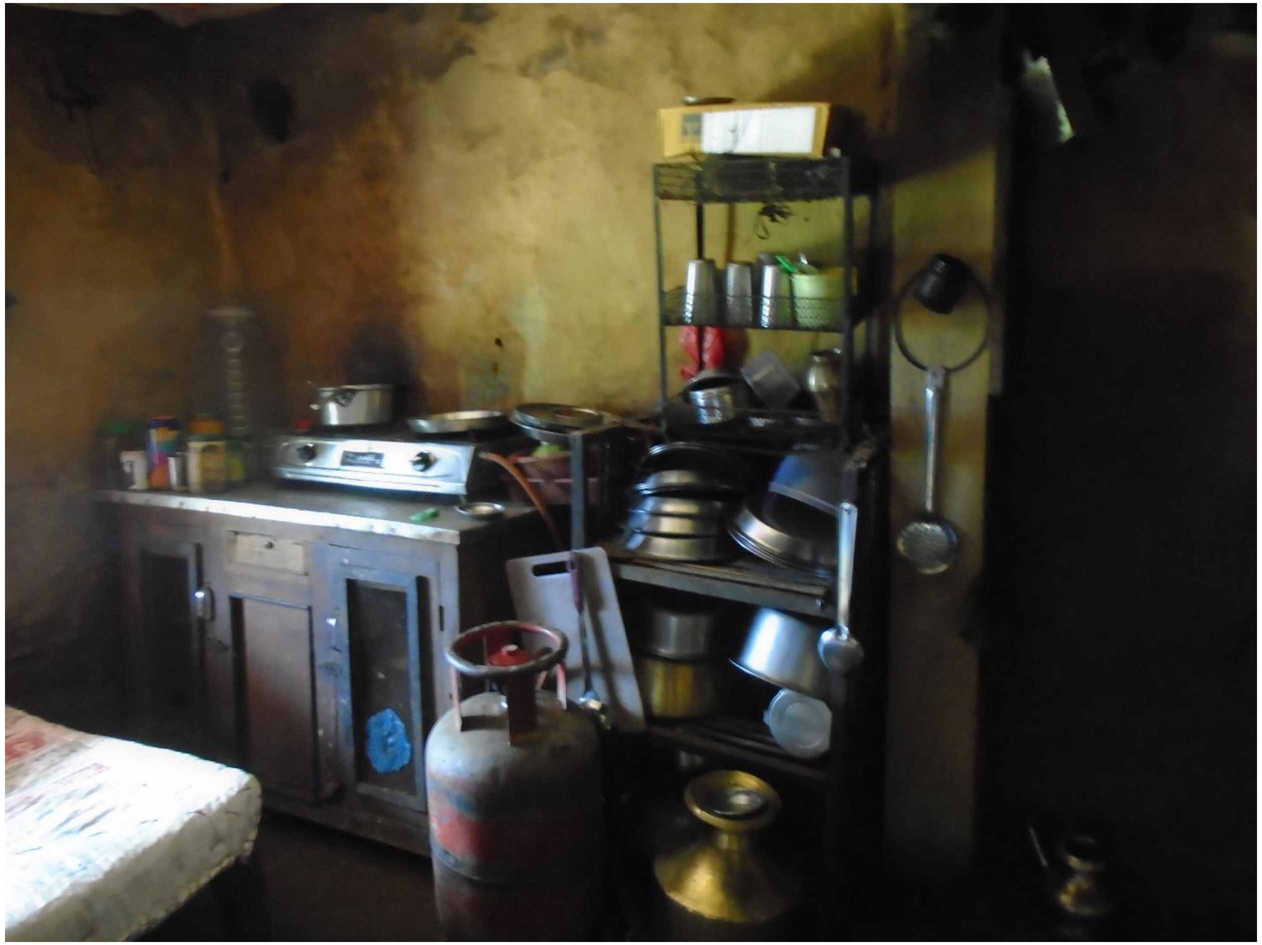

Fig. 11 "We have to follow our tradition, so should not touch kitchen during period. If one touches kitchen, worshipping area is also touched. If touched, I feel discomfort and fear that something might happen. However, in case of my daughter if she touches, god will forgive her." PhotoVoice image taken by Bishnu Maya Sapkota. Ranked 2 of 4.
