# Supplementary material for: Qualitative study exploring the barriers to menstrual hygiene management faced by adolescents and young people with a disability, and their carers in the Kavrepalanchok district, Nepal
Source: BMC Public Health. 2021 Mar 10;21:476. doi: 10.1186/s12889-021-10439-y (PMC7944905; doi:10.1186/s12889-021-10439-y)
Supplement: Supplementary file 5 — Additional file 5. Menstrual product preference across impairment type, containing Table 1: Most preferred product according to the person’s impairment, and Table 2. Least preferred product according to the person’s impairment. [file 12889_2021_10439_MOESM5_ESM.docx]

| Table 1. **Most preferred product according to the person’s impairment** | | |
| --- | --- | --- |
| **Impairment** | **Product** | **n=** |
| Hearing | Reusable pad with wings | 1 |
| Cognition | Disposable pad with wings | 1 |
| Visual | Reusable pad with wings | 1 |
|  | Disposable pad with wings | 1 |
|  | Cloth | 1 |
| Self-care | Disposable pad without wings | 1 |
| Mobility | Disposable pad with wings | 3 |
|  | Cloth | 1 |
|  | Disposable pad without wings | 1 |
| Mobility and self-care | Disposable pad with wings | 1 |
|  | Reusable pad with wings | 1 |
|  | Cloth | 1 |
| Mobility and communicating | Disposable pad with wings | 1 |
| Mobility, cognition | Disposable pad with wings | 1 |
| Totals |  | 16 |

| **Table 2. Least preferred product according to the person’s impairment*** | | |
| --- | --- | --- |
| **Impairment** | **Product** | **n=** |
| Visual | Cloth | 1 |
|  | Disposable pad without wings | 1 |
|  | Reusable pad with wings | 1 |
| Self-care | Cloth | 1 |
| Mobility | Nappy | 1 |
|  | Cloth | 1 |
|  | Disposable pad without wings | 2 |
|  | Reusable pad with wings | 1 |
| Mobility and self-care | Reusable pad with wings | 2 |
|  | Cloth | 1 |
| Mobility and communicating | Cloth | 1 |
| Totals |  | 13 |

*No data not available for three participants.
